# Quantification of spatial and phenotypic heterogeneity in an agent-based model of tumour-macrophage interactions

**DOI:** 10.1371/journal.pcbi.1010994

**Published:** 2023-03-27

**Authors:** Joshua A. Bull, Helen M. Byrne

**Affiliations:** 1 Wolfson Centre for Mathematical Biology, Mathematical Institute, University of Oxford, Oxford, United Kingdom; 2 Ludwig Institute for Cancer Research, Nuffield Department of Medicine, University of Oxford, Oxford, United Kingdom; Queensland University of Technology, AUSTRALIA

## Abstract

We introduce a new spatial statistic, the weighted pair correlation function (wPCF). The wPCF extends the existing pair correlation function (PCF) and cross-PCF to describe spatial relationships between points marked with combinations of discrete and continuous labels. We validate its use through application to a new agent-based model (ABM) which simulates interactions between macrophages and tumour cells. These interactions are influenced by the spatial positions of the cells and by macrophage phenotype, a continuous variable that ranges from anti-tumour to pro-tumour. By varying model parameters that regulate macrophage phenotype, we show that the ABM exhibits behaviours which resemble the ‘three Es of cancer immunoediting’: Equilibrium, Escape, and Elimination.

We use the wPCF to analyse synthetic images generated by the ABM. We show that the wPCF generates a ‘human readable’ statistical summary of where macrophages with different phenotypes are located relative to both blood vessels and tumour cells. We also define a distinct ‘PCF signature’ that characterises each of the three Es of immunoediting, by combining wPCF measurements with the cross-PCF describing interactions between vessels and tumour cells. By applying dimension reduction techniques to this signature, we identify its key features and train a support vector machine classifier to distinguish between simulation outputs based on their PCF signature. This proof-of-concept study shows how multiple spatial statistics can be combined to analyse the complex spatial features that the ABM generates, and to partition them into interpretable groups.

The intricate spatial features produced by the ABM are similar to those generated by state-of-the-art multiplex imaging techniques which distinguish the spatial distribution and intensity of multiple biomarkers in biological tissue regions. Applying methods such as the wPCF to multiplex imaging data would exploit the continuous variation in biomarker intensities and generate more detailed characterisation of the spatial and phenotypic heterogeneity in tissue samples.

## Author summary

Multiplex images provide exquisitely detailed information about the spatial distribution and intensity of up to 40 biomarkers within two-dimensional tissue regions, creating challenges and opportunities for quantitative analysis. Although stain intensities are measured on a continuous scale, they are typically converted into discrete labels to simplify subsequent spatial analysis. In this paper we propose a new spatial statistic, the weighted pair correlation function (wPCF), which exploits, rather than neglects, the continuous variation in stain intensity contained in multiplex images, and can characterise both spatial and phenotypic heterogeneity.

As proof-of-principle, we apply the wPCF to synthetic data that resemble multiplex images of solid tumours. We generate data from an agent-based model (ABM) that simulates macrophage-tumour interactions. The wPCF shows how the continuous label describing macrophage phenotype is spatially related to categorical labels associated with tumour cells and blood vessels. We demonstrate that correlation functions can categorise spatial relationships in a manner which is interpretable and quantitative.

The methods we present can be used to analyse both ABM simulations and multiplex imaging data, with applications that go beyond macrophage phenotype to include other biological processes that exhibit continuous variation (e.g., cancer cell stemness, biomarkers for T-cell exhaustion, and levels of oxygenation).

## Introduction

Tumours are highly heterogeneous structures, containing diverse populations of tumour cells, blood vessels, stromal cells and immune cells. The immune landscape within solid tumours is complex and varied [1, 2], with both innate and adaptive immune cells implicated in pro- and anti-tumour responses [3]. For example, high densities of tumour associated macrophages have been associated with poor prognosis in breast, prostate and head and neck cancer and with good prognosis in colorectal and gastric cancer [4, 5]. These differences may be due to the relative numbers of pro-tumour (‘M_1_’) and anti-tumour (‘M_2_’) macrophages in these cancers, but they may also be due to their morphology and spatial distribution [6–9]. For example, in non-small cell lung cancer, high infiltration rates of M_1_ macrophages into tumour islets, but not tumour stroma, have been associated with increased patient survival [10].

While macrophages are often classified as either M_1_ or M_2_, individual macrophages may exhibit a variety of behaviours. Further, their overall behaviour, or phenotype, may change over time in response to multiple microenvironmental cues [11]. Macrophage phenotype is often defined in terms of expression levels of multiple functional markers such as CD68, CD163, CD204 and CD206 [9]. It is difficult to resolve this level of heterogeneity using traditional immunohistochemistry (IHC), which typically permits only one or two markers per image. By contrast, multiplex imaging modalities, such as multiplexed IHC and imaging mass cytometry (IMC), can map expression levels of up to 40 different cellular markers and, as such, resolve the spatial position and phenotype of individual cells, including macrophages [7, 12–14].

**Fig 1:**
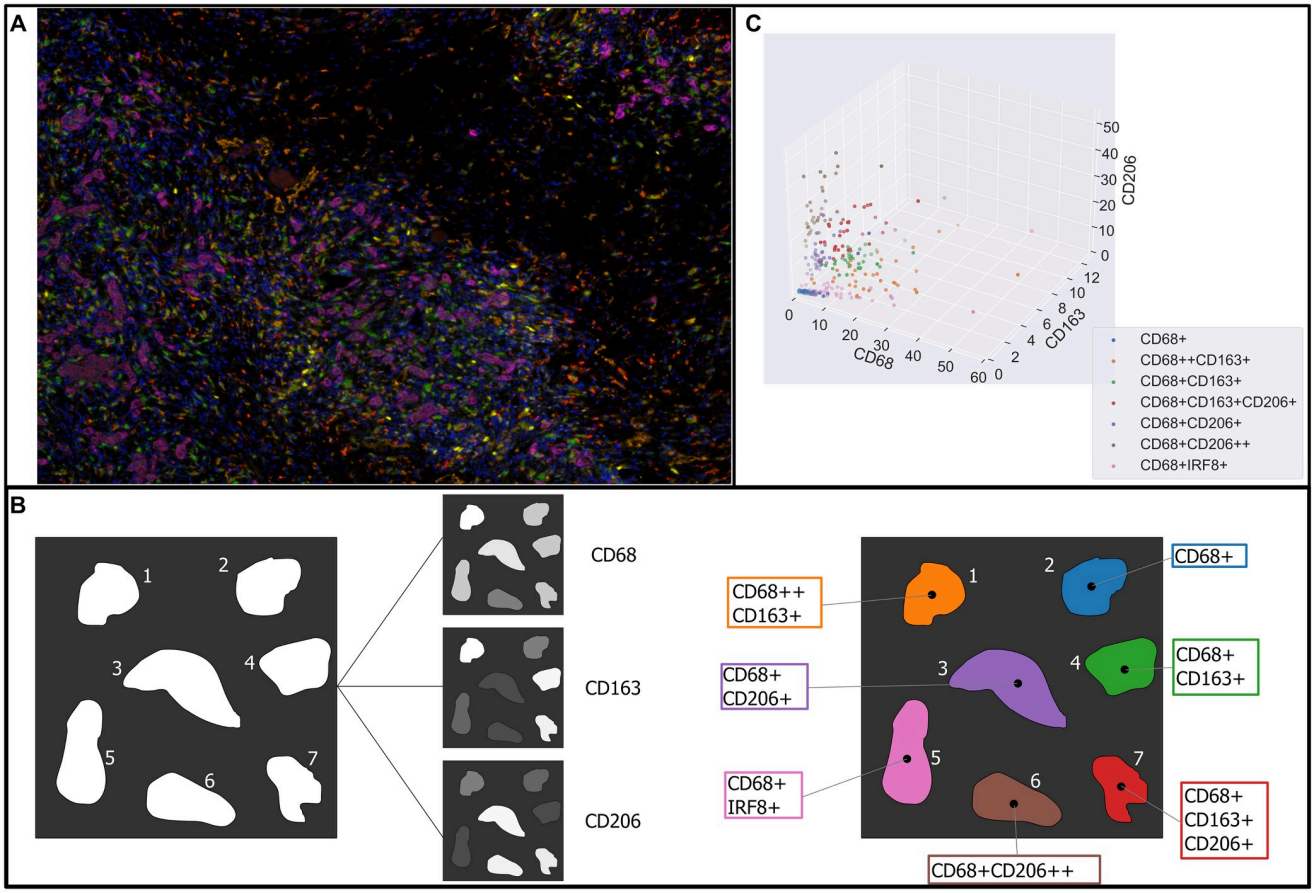
Typical process for analysing macrophage phenotypes in multiplex imaging. A: A multiplex image showing macrophages of varying phenotypes, reproduced from Fig 1b in [12]. Expression levels of different functional markers (e.g., CD68, CD163, CD206) are shown as differing intensities of separate stains, visualised as a false-colour image. (Key: Orange: CD68+CD206++, Brown: CD68+CD206+, Green: CD68+, Yellow: CD68+IRF8+, Dark red: CD68++CD163+, Red: CD68+CD163+, and Purple: CD68+CD163+CD206+). B: Schematic indicating how continuous stain intensities represented in a multiplex image are converted into different categories indicating macrophage phenotype. In this example, cell colours in the multiplex image are converted into stain intensities for CD68, CD163 and CD206. Thresholds are assigned to each stain to distinguish whether a cell is negative, positive, or extremely positive, for each stain. Each macrophage is then assigned one of 7 different potential phenotypes, based on combinations of positivity or negativity for each stain. C: Data reproduced from Fig 1h in [12]. Points represent the average stain intensity of CD68, CD163, and CD206 measured in macrophages assigned to each of the seven phenotype subtypes in each of n = 35 patients. Note that macrophages from the same subtype in different patients have differing levels of intensity of each marker, so the same categorical label may be applied to macrophages with a wide range of continuous expression levels. Fig 1 contains elements adapted from Fig 1 of https://doi.org/10.1038/s41467–019-11788-4, which is published under a Creative Commons Attribution 4.0 International License.

In Fig 1A we present a typical multiplex image which shows spatial variation in the intensity levels of three macrophage markers (CD68, CD163 and CD206), reproduced from [12]. In Fig 1B we show how the average intensity levels of these markers are used to classify segmented cells. Defined threshold intensities are used to determine whether cells are negative (-), positive (+) or extremely positive (++) for a particular marker. The classifications are combined to identify seven macrophage subtypes: CD68+, CD68++CD163+, CD68+CD163+, CD68+CD163+CD206+, CD68+CD206+, CD68+CD206++, and CD68+IRF8+. By associating one of these classifications with the centroid of each macrophage, a spatial map of macrophage subtypes can be generated and used for subsequent spatial analyses. Such analyses may include correlations of cell densities, measurements of distances between cells, or more complex spatial statistics such as pair correlation functions (PCFs) which account for spatial relations between points [15]. Fig 1C shows how the average intensity levels of CD68, CD163 and CD206 associated with each macrophage subtype vary across *n* = 35 patients (data reproduced from [12]). Variation in marker levels occurs both within a tissue sample and across patients.

Stratification of macrophage populations into discrete classes (e.g., M_1_ and M_2_ classes, or the seven subtypes shown in Fig 1), neglects the full range of information available from multiplex images. In this paper, we show how resolving continuous variation in cell labels clarifies the relationship between macrophage phenotype and spatial heterogeneity in solid tumours. To achieve this, we introduce the weighted pair correlation function (wPCF), a new spatial statistic which accounts for continuous variation in labels such as cell subtype, phenotype, or marker expression levels. While similar ‘marked point patterns’ have been studied in ecology [16–18] and astronomy [19, 20], few methods consider the spatial correlation of continuous marks. Existing methods, such as Stoyan’s mark correlation function [16, 19, 21, 22] or the mark variogram [18, 23], typically only depend on the distance *r* between point pairs and quantify the correlation between marks at distance *r*. For example, the mark correlation function *k*_*mm*_ determines whether the marks of two points separated by a distance *r* are spatially correlated: *k*_*mm*_(*r*) > 1 indicates that the marks of points separated by *r* are larger than the average mark, while *k*_*mm*_(*r*) < 1 indicates that they are smaller than the average [16]. The mark variogram can be used to test the similarity of two marks, separated by a distance *r* [18, 23]. While these methods are powerful, they are designed to evaluate the similarity of marks on points separated by a fixed distance, rather than to consider the spatial correlation of points with specified marks. In biological applications, a relevant problem is to determine whether points with a particular mark, or range of marks, are correlated at distance *r*. The wPCF addresses this, by identifying spatial interactions between points of one type and those whose mark is within a range of target values.

We test and validate the wPCF using synthetic data generated from a two-dimensional agent-based model (ABM) of tumour growth that accounts for tumour-macrophage interactions and dynamic changes in macrophage phenotype. ABMs are well-suited for generating labelled point pattern data, since each cell is represented by an agent whose behaviour is determined by subcellular variables (describing, for example, cell cycle state [24] or phenotype), and its interaction with the environment (e.g., through force laws describing mechanical interactions between cells). These subcellular variables can be used to represent marker expression levels, meaning that each agent is associated with a point representing its cell centre together with a collection of continuous or categorical labels. Data from such ABMs can be analysed using PCFs [25–30] or cross-PCFs [31, 32], an extension of the PCF which accounts for interactions between cells of different types.

The off-lattice, force-based ABM that we develop is motivated by an experimental study by Arwert *et al* [33] which investigates how macrophage phenotype depends on spatial location relative to a tumour mass and nearby vasculature, and how the spatial distribution of the different macrophage phenotypes influences the tumour’s growth dynamics. In brief, anti-tumour macrophages extravasate from blood vessels and migrate towards clusters of tumour cells, in response to tumour-derived signals such as colony stimulating factor-1 (CSF-1). Exposure to TGF-*β* in the tumour increases macrophage sensitivity to C-X-C chemokine ligand type 12 (CXCL12) and drives them towards a pro-tumour phenotype. At the same time, CXCL12 produced by perivascular fibroblasts biases the movement of these M_2_ macrophages towards neighbouring blood vessels. As they migrate out of the tumour, the pro-tumour macrophages express epidermal growth factor (EGF), a tumour cell chemoattractant. In this way, M_2_ macrophages facilitate metastasis by guiding the tumour cells towards the vasculature [33, 34].

The hybrid ABM developed in this paper builds on existing differential equation models [35, 36] and ABMs [36, 37] that focus on specific tumour-macrophage interactions, such as the CSF-1/EGF paracrine loop that mediates cross-talk between tumour cells and macrophages. Our model accounts for macrophage extravasation in response to tumour-derived CSF-1, their subsequent tumour infiltration, and the CSF-1/EGF paracrine loop that mediates cross-talk between tumour cells and macrophages. Models of macrophage-tumour interactions often view macrophages as a homogeneous population [38, 39], or account for multiple macrophage subtypes (typically M_1_ and M_2_) [40–43] and their interactions with T-cells [44–46]. Eftimie [47] and El-Kenawi *et al*. [48] have developed models that view macrophage phenotype as a continuous variable whose dynamics are governed by environmental cues, such as pH. We represent macrophage phenotype as a continuous variable, *p*, whose dynamics depend on local levels of TGF-*β* and determine macrophage behaviour.

We vary ABM parameters, and show that it generates a range of spatial patterns and qualitative behaviours that resemble ‘The Three Es of cancer immunoediting’ [49]. In particular, as we vary model parameters associated with the rate of macrophage extravasation and their chemotactic sensitivity to CSF-1, we observe tumour Elimination, Escape, and Equilibrium. We analyse simulation outputs at a fixed timepoint using wPCFs and cross-PCFs. We explain how, taken together, wPCFs and cross-PCFs provide a description of simulation outputs which is both quantitative and interpretable. We then show how the spatial statistics can be combined and analysed using principal component analysis (PCA) to identify the key features that characterise Elimination, Escape, and Equilibrium for our ABM simulations. We show further how the principal components can be used to classify unseen data from ABM simulations. More generally, this study serves as a powerful proof of concept: it shows how combinations of wPCFs and cross-PCFs could be used to accurately classify the complex spatial and phenotypic patterns formed by cells in multiplex images, without manual thresholding of marker intensities.

The remainder of the paper is structured as follows. We first describe our ABM, emphasising those features which make it appropriate for generating synthetic imaging data. We introduce the wPCF and illustrate how it can be interpreted as a series of cross-PCFs which vary continuously with the label of interest, here macrophage phenotype. We then apply the wPCF to synthetic data generated from our ABM, and show that it provides a more detailed description of the relationship between macrophage phenotype and spatial location than cross-PCFs. We define a ‘PCF signature’, consisting of two wPCFs and a cross-PCF, which describes the spatial relationships between blood vessels, tumour cells, and macrophages of each phenotype. The signature can be interpreted as a high-dimensional vector, and we apply principal component analysis (PCA) to reduce its dimensionality. We demonstrate a proof-of-concept classification algorithm by using the first 100 principal components to train a simple classifier which distinguishes between simulation outputs that correspond to the three Es of immunoediting (i.e., Escape, Equilibrium and Elimination). Finally, we calculate PCF signatures for dynamic ABM data, and show that a single simulation may transition between tumour Equilibrium, Escape and Elimination at different timepoints.

## Materials and methods

### Agent-based model (ABM)

We present a 2D, multiscale, off-lattice ABM which extends an existing model of macrophage infiltration into tumour spheroids by accounting for continuous and dynamic variation in macrophage phenotype [30, 50]. The new model simulates a growing tumour embedded in a small tissue region *in vivo*, and includes phenotype-dependent interactions between macrophages, blood vessels and tumour cells. We outline the ABM here, and refer the interested reader to S1 Appendix for details of the implementation and default parameter values. The ABM is implemented within the open source Chaste (Cancer, Heart and Soft Tissue Environment) modelling environment [51–53].

#### Overview

**Fig 2:**
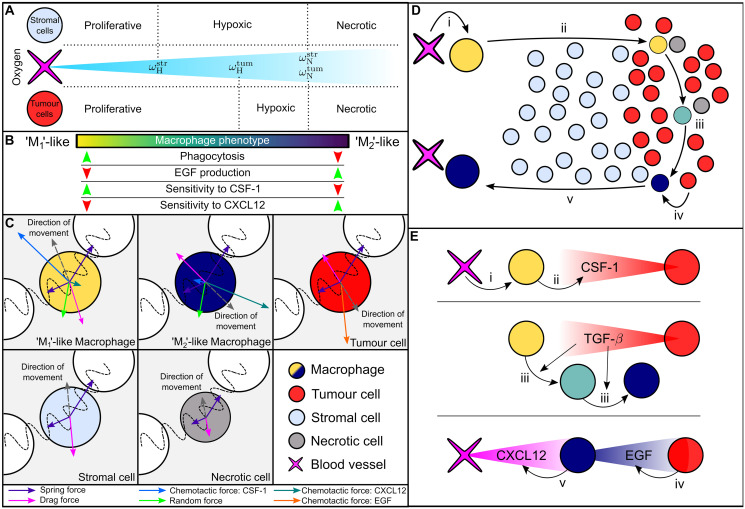
Schematic summarising the key interactions that are included in the agent based model. A: Oxygen is supplied by blood vessels and consumed by stromal cells and tumour cells. Cell-cycle progression is determined by a cell’s local oxygen concentration: a cell may be ‘proliferative’ (and progress through its cell cycle), ‘hypoxic’ (the cell cycle is temporarily paused until oxygen concentrations return to a sufficiently high level) or ‘necrotic’ (the cell becomes necrotic cell and degrades). Cell cycles also pause if there is insufficient space available for proliferation. B: Macrophage behaviour depends on phenotype *p*, modulating their rates of tumour cell killing, EGF production, and chemotactic sensitivity to gradients of CSF-1 and CXCL12. C: Forces acting on different cell types. Macrophages are subject to mechanical forces due to interactions with nearby cells, and random forces which simulate their exploration of their environment as highly motile cells. Macrophages also experience chemotactic forces that are directed up spatial gradients of CSF-1 and CXCL12, and whose magnitude depends on *p*. Tumour cells experience mechanical forces due to interactions with neighbouring cells, and chemotactic forces in the direction of increasing EGF. Stromal cells experience mechanical forces due to interactions with neighbouring cells. Necrotic cells experience these interaction forces, which decrease in magnitude as they decrease in size. All cells experience a drag force. D: Summary of the phases of macrophage-mediated tumour cell migration in our ABM. i) M_1_ macrophages extravasate from blood vessels in response to CSF-1. ii) M_1_ macrophages migrate into the tumour mass in response to CSF-1, where they may kill tumour cells. iii) Exposure to TGF-*β* causes macrophages to adopt an M_2_ phenotype. iv) M_2_ macrophages produce EGF, which acts as a chemoattractant for tumour cells. v) M_2_ macrophages migrate towards blood vessels, in response to CXCL12 gradients. E: Schematic summarising the sources of CSF-1, TGF-*β*, EGF and CXCL12 in our model, and their interactions with cells, as described in steps i-v of panel D.

The ABM distinguishes four cell types: stromal cells, tumour cells, necrotic cells, and macrophages. Their dynamics are influenced by five diffusible species: oxygen (*ω*), CSF-1 (*c*), CXCL12 (*ξ*), TGF-*β* (*g*) and EGF (*ϵ*). In the 2D Cartesian geometry, blood vessels are represented by fixed points which do not compete for space with the cell populations and which act as distributed sources of oxygen [54]. A schematic of the ABM is presented in Fig 2.

Following [55], critical oxygen thresholds for hypoxia ($\omega_{H}^{\text{str}}$ and $\omega_{H}^{\text{tum}}$) and necrosis ($\omega_{N}^{\text{str}}$ and $\omega_{N}^{\text{tum}}$) relate the rates of cell cycle progression of stromal and tumour cells to local oxygen levels (see Fig 2A for details). For example, if, at time *t* > 0, $\omega_{N}^{\text{tum}}<\omega \left(x,t\right)<\omega_{H}^{\text{tum}}$, then the cell cycle of a tumour cell at position **x** will immediately halt and remain paused until the local oxygen concentration rises above the tumour hypoxic threshold. If, however, the oxygen concentration falls below the tumour necrosis threshold, then the cell becomes necrotic (this switch is irreversible). Necrotic cells occupy space for a finite time period during which their size decreases linearly to zero and they are then removed from the simulation. Blood vessels also act as entry points for macrophages, which infiltrate the tissue and alter their phenotype (and, hence, behaviour) at rates which depend on local levels of TGF-*β* (see Fig 2B).

We represent each cell by the spatial coordinates of its centre of mass and determine its movement by balancing the forces that act on it. Using an overdamped form of Newton’s second law and neglecting inertial terms, we have that for cell *i*

$$\begin{array}{c} \nu \frac{dx_{i}}{dt}=F_{i}, \end{array}$$ (1)

where *ν* is the assumed constant drag coefficient and **F**_*i*_ denotes the net force acting on cell *i* at position **x**_*i*_ and time *t*. The forces that act on a cell depend on its type (see Fig 2C and S1 Appendix). Cells interact via spring forces if their centres are within a distance *R*_int_ of each other [56]; intercellular adhesion and volume exclusion are represented by attractive and repulsive forces respectively. We also associate with each cell an approximate area, and stromal cells which are so mechanically compressed that their area falls below a threshold proportion $A_{i\;\text{str}}^{H}$ of their normal area pause their cell cycle due to contact-inhibition (see S1 Appendix for details).

#### Macrophage phenotype

Since the experimental data shown in [33] describes a unidirectional transition of macrophage phenotype, we use a single continuous subcellular variable to represent macrophage phenotype. This variable, *p* ∈ [0, 1], determines macrophage behaviour, with *p* < 0.5 denoting a primarily anti-tumour M_1_ phenotype and *p* > 0.5 representing a primarily pro-tumour M_2_ phenotype. While M_1_ and M_2_ describe two broad categories of macrophage, their underlying behaviour is dependent on the value of *p* rather than this categorisation, and for simplicity we may refer to macrophages whose phenotype is close to 0.5 as ‘transitioning’ macrophages. We assume that, following extravasation, macrophage *i* has a phenotype *p*_*i*_ = 0. Macrophage exposure to TGF-*β* levels above a threshold value, *g*_crit_, causes *p*_*i*_ to increase at a constant rate Δ*p*, per timestep *dt*, until the maximum value *p*_*i*_ = 1 is reached and the macrophage has a fully M_2_ phenotype. Its phenotype remains fixed at *p*_*i*_ = 1 for all later times. Thus, we have:

$$\begin{array}{c} \frac{dp_{i}}{dt}=H\left(g\left(x_{i},t\right)-g_{\text{crit}}\right)H\left(1-p_{i}\right)\Delta p, \end{array}$$ (2)

where H is the Heaviside function (H(x)=1 when *x* > 0 and H(x)=0 otherwise).

We now explain how changes in phenotype *p* affect macrophage behaviour and function, and how these changes are incorporated into the ABM (see also Fig 2B).

#### Macrophage extravasation

Macrophages enter the domain via blood vessels with a probability per hour *P*_ex_ which is an increasing, saturating function of CSF-1:

$$\begin{array}{c} P_{\text{ex}}=P^{⋆}\times \frac{c}{c+c_{1/2}}, \end{array}$$ (3)

where the non-negative parameter *P*^⋆^ represents the maximum probability per hour of macrophage extravasation from a vessel, and *c*_1/2_ is the concentration of CSF-1 at which the probability is half-maximal.

#### Macrophage chemotactic forces

Fig 2C shows the forces which act on different cell types (functional forms for these forces are given in S1 Appendix). Here we highlight two macrophage-specific forces which describe their directed movement up spatial gradients of CSF-1 and CXCL12, and whose magnitude varies with phenotype *p*. Noting that M_1_ macrophages are sensitive to CSF-1 and insensitive to CXCL12 (and conversely for M_2_ macrophages), we assume that chemotactic forces depend linearly on phenotype. The chemotactic forces acting on macrophage *i*, at position **x**_*i*_ with phenotype *p*_*i*_, are therefore:

$$\begin{array}{c} F_{i}^{\chi_{c}}=\chi_{c}^{m}\left(1-p_{i}\right)\frac{\nabla c}{\left|\nabla c\right|}\;\text{and}\;F_{i}^{\chi_{\xi }}=\chi_{\xi }^{m}p_{i}\frac{\nabla \xi }{\left|\nabla \xi \right|} \end{array}$$ (4)

respectively, where the non-negative parameters $\chi_{c}^{m}$ and $\chi_{\xi }^{m}$ indicate macrophage sensitivity to spatial gradients of CSF-1 and CXCL12, and ∇*c* and ∇*ξ* are evaluated at **x**_*i*_. The forces $F_{i}^{\chi_{c}}$ and $F_{i}^{\chi_{\xi }}$ contribute to the net force **F**_*i*_ in Eq (1) (see Fig 2C and S1 Appendix).

#### Macrophage cell killing

We assume that when a macrophage and a tumour cell are within the interaction radius *R*_int_ then the macrophage will attempt to kill the tumour cell, with M_1_ macrophages more likely to kill a tumour cell than M_2_ macrophages. We define a probability of cell kill per hour, *P*_*φ*_, which is a monotonic decreasing function of *p*. We suppose further that, after a macrophage has killed a tumour cell, it experiences a ‘cooldown’ period of *t*_cool_ hours during which it cannot attempt tumour cell killing. Thus, we associate with macrophage *i* a subcellular timer *t*_*φ*,*i*_ that is updated in real time and set to zero on tumour cell killing. We define *P*_*φ*,*i*_ as:

$$\begin{array}{c} P_{\varphi }{}_{,i}=\{\begin{array}{cc} P_{\varphi }^{⋆}\times \left(1-\frac{p_{i}^{10}}{p_{i}^{10}+0.5^{10}}\right) & \text{for}\;t_{\varphi }{}_{,i}\geq t_{\text{cool}}\\ 0 & \text{otherwise} \end{array}, \end{array}$$ (5)

where $P_{\varphi }^{⋆}$ is the maximum probability of tumour cell killing. If macrophage *i* is sufficiently close to attack multiple tumour cells then one is selected at random for cell death. Killed tumour cells are labelled as ‘necrotic’ and decay in the same way as other necrotic cells.

#### Macrophage production of EGF

The diffusible cytokine EGF, *ϵ*, is produced by M_2_ macrophages and undergoes natural decay. It is also a potent chemoattractant for tumour cells. For simplicity, we assume that macrophage *i* produces EGF at a rate which is linearly proportional to its phenotype *p*_*i*_, with constant of proportionality *κ*_*ϵ*_. Denoting by *D*_*ϵ*_ and λ_*ϵ*_ the assumed constant diffusion coefficient and natural decay rate of EGF, we suppose that its evolution can be described by the following reaction diffusion equation:

$$\begin{array}{c} \frac{\partial \epsilon }{\partial t}=D_{\epsilon }\nabla^{2}\epsilon -\lambda_{\epsilon }\epsilon +\kappa_{\epsilon }\sum_{i}p_{i}\delta \left(x-x_{i}\right). \end{array}$$ (6)

where *δ*(**x**) = 1 when **x** = 0 and *δ*(**x**) = 0 elsewhere. In (6), we sum over all macrophages to determine the net rate of production at spatial position **x**.

### Spatial statistics

In order to compute spatial statistics, we introduce the following notation. Consider an object *i* (which may be a cell or a blood vessel), whose centre is located at **x**_*i*_ = (*x*_*i*_, *y*_*i*_) at time *t*. We associate with object *i* a categorical label *q*_*i*_ ∈ {*B*, *M*, *S*, *T*, *N*} which indicates whether it is a blood vessel, macrophage, stromal cell, tumour cell or necrotic cell. Given a target label *Q* ∈ {*B*, *M*, *S*, *T*, *N*}, the binary target function Θ(*Q*, *q*_*i*_) indicates whether the label *q*_*i*_ matches *Q*:

$$\begin{array}{c} \Theta \left(Q,q_{i}\right)=\{\begin{array}{cc} 1 & \text{if}\;q_{i}=Q,\\ 0 & \text{otherwise}. \end{array} \end{array}$$ (7)

#### Cross pair correlation function (cross-PCF)

The cross-PCF identifies spatial correlations between objects with categorical labels that are separated by a distance *r*. We define a sequence of annuli, of inner radius *r*_*k*_ and outer radius *r*_*k*_ + *dr* where *r*_0_ = 0 and *dr* > 0. We denote by $A_{r_{k}}\left(x\right)$ the area of the annulus with inner radius *r*_*k*_ that is centred at the point **x**. If this annulus lies wholly inside the domain then $A_{r_{k}}\left(x\right)=\pi \left({\left(r_{k}+dr\right)}^{2}-r_{k}^{2}\right)=\pi \left(2r_{k}+dr\right)dr$; otherwise, only the area contained within the domain is recorded. The indicator function, *I*_*k*_(*r*), is defined as follows:

$$\begin{array}{c} I_{k}\left(r\right)=\{\begin{array}{cc} 1 & \text{for}\;r_{k}\leq r<r_{k}+dr,\\ 0 & \text{otherwise}. \end{array} \end{array}$$ (8)

We calculate the cross-PCF for blood vessels and tumour cells by considering a region of area *A*. If, at time *t*, this region contains *N*_*B*_ blood vessels and *N*_*T*_ tumour cells, then the cross-PCF, *g*_*BT*_(*r*), is given by:

$$\begin{array}{cc} g_{BT}\left(r\right) & =\frac{1}{N_{B}}\sum_{i=1}^{N}\Theta \left(B,q_{i}\right)(\sum_{j=1}^{N}\Theta \left(T,q_{j}\right)\frac{I_{k}\left(\right|x_{i}-x_{j}\left|\right)}{A_{r_{k}}\left(x_{i}\right)}/\frac{N_{T}}{A})\\ \Rightarrow g_{BT}\left(r\right) & =\frac{1}{N_{B}N_{T}}\sum_{i=1}^{N}\sum_{j=1}^{N}\frac{A}{A_{r_{k}}\left(x_{i}\right)}\Theta \left(B,q_{i}\right)\Theta \left(T,q_{j}\right)I_{k}\left(\right|x_{i}-x_{j}\left|\right). \end{array}$$ (9)

where *r* ∈ [*r*_*k*_, *r*_*k*_ + *dr*) and *N* is the total number of objects in the simulation. For each blood vessel, the cross-PCF compares the density of tumour cells in the annulus that surrounds it to *N*_*T*_/*A*, the expected density in the annulus under complete spatial randomness (CSR). Thus, *g*_*BT*_(*r*) > 1 indicates clustering of tumour cells at distance *r* from blood vessels and *g*_*BT*_(*r*) < 1 indicates anti-correlation, or exclusion, of tumour cells at distance *r* from blood vessels. Cross-PCFs for other pairs of categorical variables are defined similarly. We note that the cross-PCF is not necessarily symmetric (i.e., *g*_*BT*_ ≠ *g*_*TB*_ since, for any pair of points, the annulus surrounding one point may intersect with the domain boundary while the annulus surrounding the other may not).

#### Weighted pair correlation function (wPCF)

We calculate the wPCF by replacing Θ(*Q*, *q*_*i*_) in Eq (9) with a weighting function, 0 ≤ *w*_*p*_(*P*, *p*_*i*_) ≤ 1, which describes how *p*_*i*_ differs from a target phenotype, *P*. Multiple functional forms could be used for the weighting function. The relationship between the wPCF and cross-PCF is explored in more detail in S3 Appendix, and in S4 Appendix we show how the choice of weighting function affects the wPCF. For simplicity we use the same weighting function throughout this paper. We use a triangular weighting function of the form:

$$\begin{array}{c} w_{p}\left(P,p_{i}\right)=\text{max}(1-\frac{|P-p_{i}|}{\Delta P},0), \end{array}$$ (10)

and fix Δ*P* = 0.2. Then, *w*_*p*_ ≈ 1 for cells whose phenotype *p*_*i*_ is close to the target *P* and *w*_*p*_ = 0 for those with |*P* − *p*_*i*_| > Δ*P*. We note further that *w*_*p*_(*P*, *p*_*i*_) → Θ(*P*, *p*_*i*_) as Δ*P* → 0. The choice of Δ*P*, therefore, represents a balance between obtaining a smoothly varying wPCF (reduced noise) and ensuring that the wPCF displays the signal of interest (increased signal). Different values of Δ*P* are considered in S4 Appendix.

Replacing Θ(*T*, *q*_*i*_) with *w*_*p*_(*P*, *p*_*i*_) in Eq (9), we define the wPCF for macrophages of target phenotype *P* and blood vessels *B*, at lengthscale *r*, as follows:

$$\begin{array}{c} wPCF\left(r,P,B\right)=\frac{1}{W_{P}N_{B}}\sum_{i=1}^{N}\sum_{j=1}^{N}\frac{A}{A_{r_{k}}\left(x_{i}\right)}w_{p}\left(P,p_{i}\right)\Theta \left(B,q_{j}\right)I_{k}\left(\right|x_{i}-x_{j}\left|\right) \end{array}$$ (11)

where $W_{P}\left(P\right)=\sum_{i=1}^{N}w_{p}\left(P,p_{i}\right)$ is the total ‘weight’ associated with the target label *P* across all macrophages (*W*_*P*_(*P*) replaces *N*_*T*_ in Eq (9); non-macrophages have weight *w*_*p*_ = 0). Intuitively, the wPCF extends the cross-PCF by weighting the contribution of each macrophage based on how closely its phenotype matches the target phenotype.

**Fig 3:**
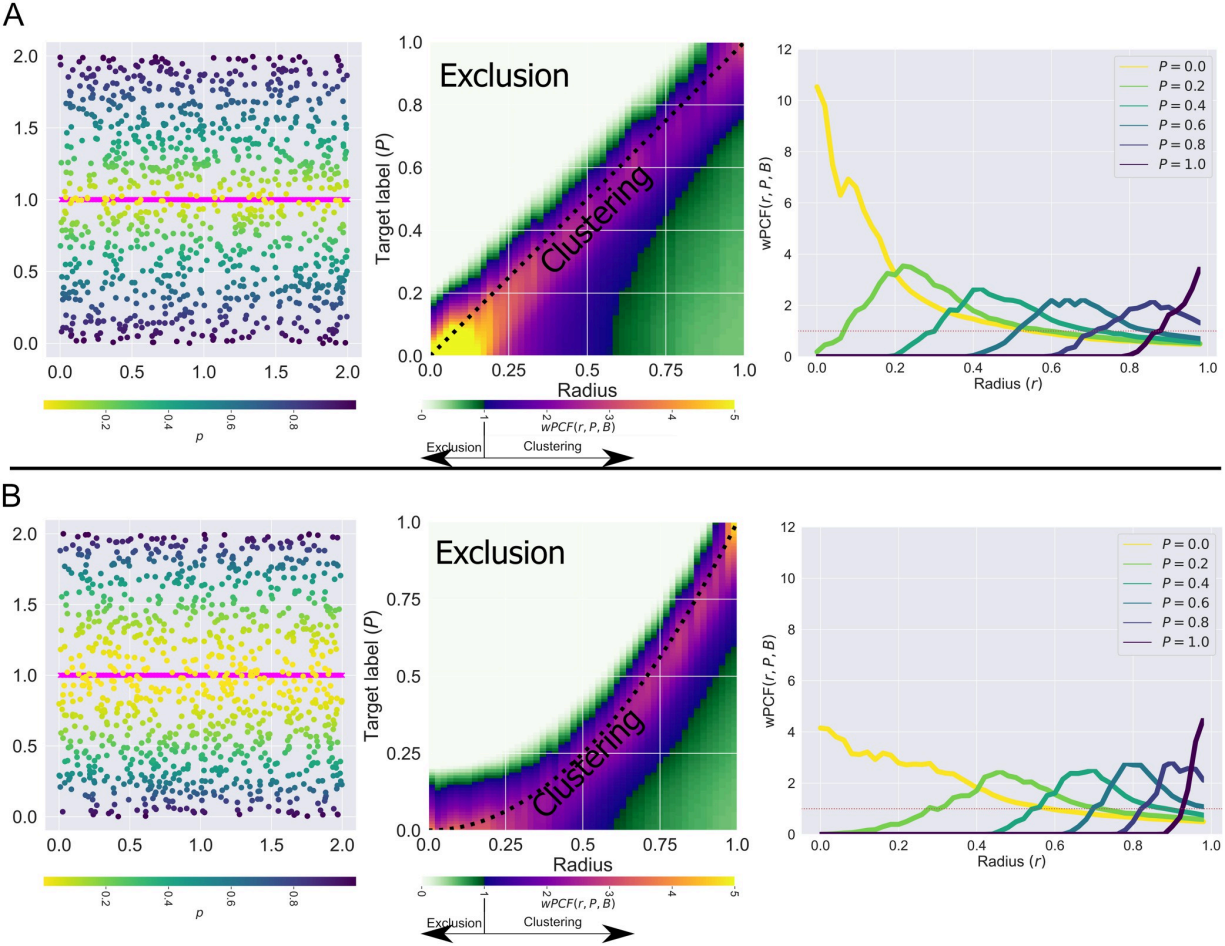
Examples for interpreting the wPCF. Two examples showing how the wPCF can identify spatial correlations between categorical objects (200 pink crosses equally spaced on the line *y* = 1, appearing as a solid line here due to the density of crosses) and objects with real values (1000 randomly placed circles with labels *p* ∈ [0, 1]). A: Points are labelled according to the formula *p*_*i*_ = |1 − *y*_*i*_|. B: Points are labelled according to the formula *p*_*i*_ = |1 − *y*_*i*_|^2^. Left: Point patterns consisting of equally spaced pink crosses and randomly placed circles with non-random labelling. Middle: wPCFs corresponding to the above point patterns. Dashed black lines show the lines *P* = *r* and *P* = *r*^2^, which by construction should show the strongest correlation. Right: Horizontal slices through the wPCF at fixed values of *P*. Such slices can be interpreted as a cross-PCF showing colocalisation between the pink crosses and circles with labels close to *P*.

In Fig 3 we present two examples showing how the wPCF characterises spatial correlations between objects with a continuous label *p* (coloured circles, analogous to macrophages with phenotype *p*) and objects with a categorical label (magenta crosses, analogous to blood vessels). In both examples, 200 crosses are uniformly distributed along the line *y* = 1, and 1000 circles are randomly placed throughout a square domain of edge length 2. In Fig 3A, the label *p*_*i*_ of a circle at (*x*_*i*_, *y*_*i*_) increases linearly with distance from the line *y* = 1 (*p*_*i*_ = |1 − *y*_*i*_|); in Fig 3B, the label increases with the square of this distance (*p*_*i*_ = |1 − *p*_*i*_|^2^). The corresponding wPCFs are shown in the middle panels of Fig 3, for a range of target labels *P* and distances *r*. For simplicity, when a wPCF is calculated over multiple values of the label *p*, we denote the resulting metric as *wPCF*(*r*, *p*, *B*). By construction, a circle at distance *r* from the nearest cross has label *P* ≈ *r* for (A) (and *P* ≈ *r*^2^ for (B)). The two wPCFs show strong clustering along these lines and exclusion at shorter distances for points with larger labels (above the dashed lines). The weaker clustering observed below the lines is explained as follows. Consider a cross at position (*x*_*j*_, 1). In (A), the largest label associated with a circle at distance *r* from this cross is *p* = *r* (if the circle is directly above the cross). Smaller labels can also be recorded, for circles at distance *r* which are offset from the cross in the y-direction. In the rightmost panels of Fig 3, we plot *wPCF*(*r*, *P*, *B*) for fixed values of the target label *P*. These curves can be interpreted as cross-PCFs for points whose labels *p*_*i*_ are “close” to *P*, and show the strongest clustering at the expected values.

A natural extension to the wPCF considers objects with two continuous labels, *P*_1_ and *P*_2_, say, in order to identify spatial correlations between objects with labels close to *P*_1_ and objects with labels close to *P*_2_ (e.g., colocalisation of macrophages with *p* ≈ 0 with those with *p* ≈ 1, or colocalisation of a particular macrophage phenotype with a particular concentration of CSF-1). We discuss such extensions in S5 Appendix.

## Results

### Agent-based modelling generates patterns that resemble the 3 Es of immunoediting

**Fig 4:**
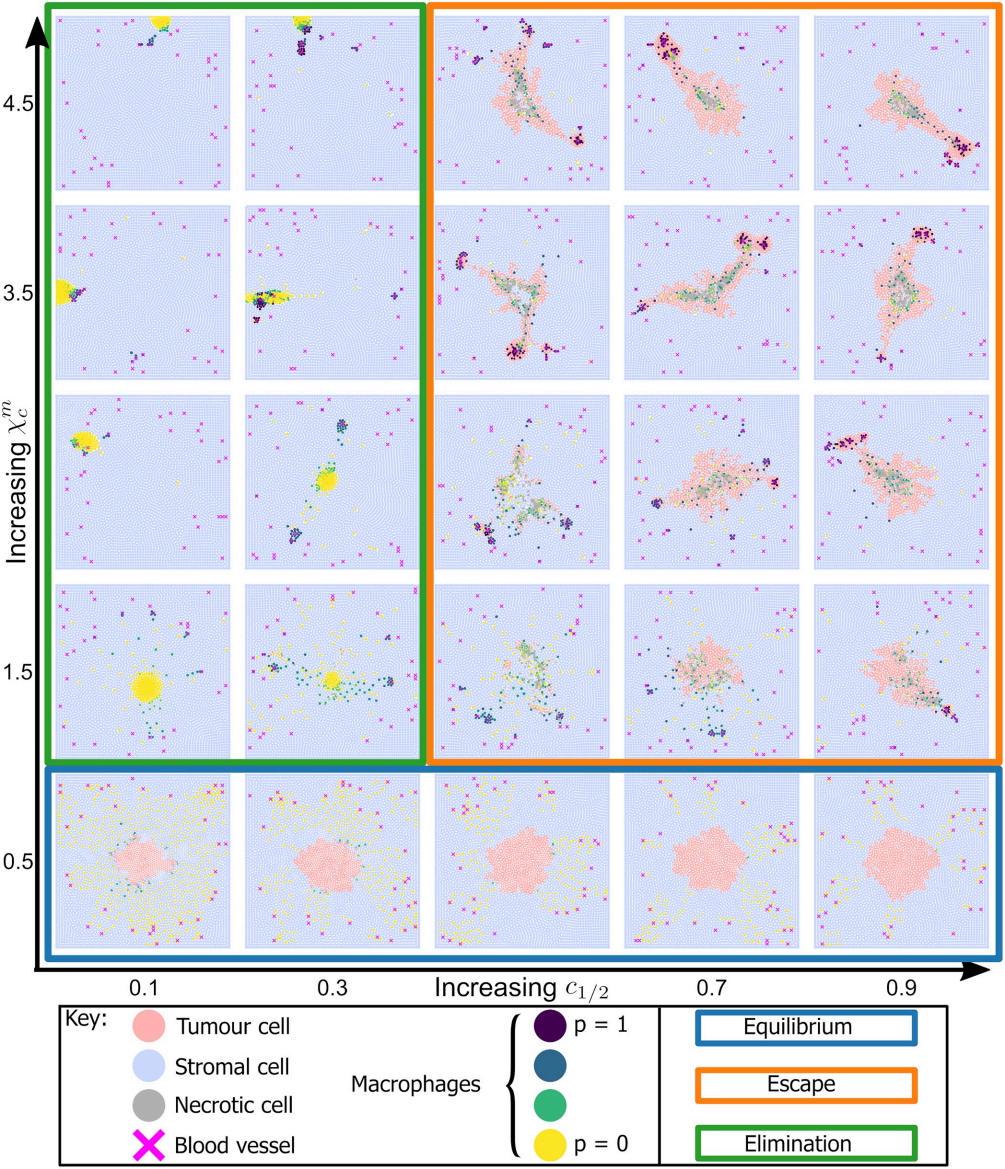
Varying macrophage sensitivity to environmental cues generates diverse patterns of tumour growth. Representative simulation endpoints for combinations of $\chi_{c}^{m}=0.5,1.5,2.5,3.5,4.5$ and *c*_1/2_ = 0.1, 0.3, 0.5, 0.7, 0.9. We group these into three qualitatively similar behaviours: Equilibrium—blue box: compact tumour mass, with predominantly M_1_ macrophages confined to the stroma. Escape—orange box: establishment of perivascular niches containing M_2_ macrophages, tumour cells and blood vessels. Tumour masses are asymmetrical. Elimination—green box: total or near total tumour elimination.

For a given set of parameter values, we run multiple realisations of the ABM and record simulation outputs at *t* = 500 (see S2 Appendix for details on simulation progression). This process generates synthetic images that resemble multiplex data, in which five categories of cells are distinguished (tumour, stroma, necrotic, vessel, macrophage) and macrophage phenotype is described using the continuous label *p*. We perform a parameter sweep of the ABM, in which we vary $\chi_{c}^{m}$, the strength of macrophage chemotaxis towards CSF-1, and *c*_1/2_, the concentration of CSF-1 at which macrophage extravasation is half-maximal, selecting values for $\chi_{c}^{m}$ and *c*_1/2_ from a discrete set of points arranged on a regular grid. We consider 9 values of each parameter, evenly spaced for $\chi_{c}^{m}\in \left[0.5,4.5\right]$ and *c*_1/2_ ∈ [0.1, 0.9]. All other parameters are held fixed at their default values (see S1 Appendix). In Fig 4 we present typical simulation outputs at *t* = 500 for different parameter combinations (some parameter combinations are omitted to facilitate visualisation). These results show that varying $\chi_{c}^{m}$ and *c*_1/2_ can generate a range of qualitative behaviours that mirror the three stages of cancer immunoediting [49]. We summarise these behaviours as follows:

- **Equilibrium**: a compact tumour mass, with macrophages confined to the surrounding stroma. The dominant macrophage phenotype is M_1_. Tumour growth is constrained, with tumour cells restricted to the mass and prevented from migrating to the vasculature by macrophage surveillance (blue box in Fig 4).
- **Escape**: the tumour has a diffuse, fragmented structure. Perivascular niches containing M_2_ macrophages and tumour cells surround blood vessels. The bulk of the tumour is infiltrated with M_1_ and transitioning macrophages, with central tumour necrosis caused by macrophages killing tumour cells (orange box in Fig 4).
- **Elimination**: total, or near-total, tumour cell elimination. Some macrophages cluster around any surviving tumour cells and the dominant macrophage phenotype is M_1_ (green box in Fig 4).

Equilibrium (blue box) arises for low values of $\chi_{c}^{m}$ (e.g, $\chi_{c}^{m}=0.5$). Large numbers of macrophages extravasate in response to CSF-1 but, since they are not strongly attracted to the tumour mass, they remain in the stroma. As a result, tumour growth is constrained by the macrophages, which are predominantly M_1_. When *c*_1/2_ is also small (e.g., *c*_1/2_ ⪅ 0.3), the rate of macrophage extravasation is high, and some macrophages reach the tumour boundary through random exploration of the tissue. These macrophages kill tumour cells on contact, causing the tumour mass to decrease in size.

Escape (orange box) occurs when macrophages migrate to the tumour so slowly that they do not overwhelm it. Further, exposure to TGF-*β* causes the macrophages to transition to an M_2_ phenotype. The M_2_ macrophages migrate towards nearby blood vessels, up spatial gradients in CXCL12, and the CSF-1/EGF paracrine loop enables tumour cells to trail behind them. If these tumour cells reach the vasculature, we assume that tumour cells enter the vasculature and metastasise to other parts of the body. Therefore, we denote such simulations as tumour escape.

Elimination (green box) occurs when the rate of macrophage extravasation is very high (low values of *c*_1/2_). Tumour elimination occurs because the M_1_ macrophages are strongly attracted to the tumour mass and kill tumour cells before they are ‘reprogrammed’ to an M_2_ phenotype. Strong chemotactic sensitivity to CSF-1 (large values of $\chi_{c}^{m}$) can cause the macrophages to cluster around the last tumour cells to be eliminated.

### The wPCF clarifies the relationship between macrophage phenotype and spatial distribution

**Fig 5:**
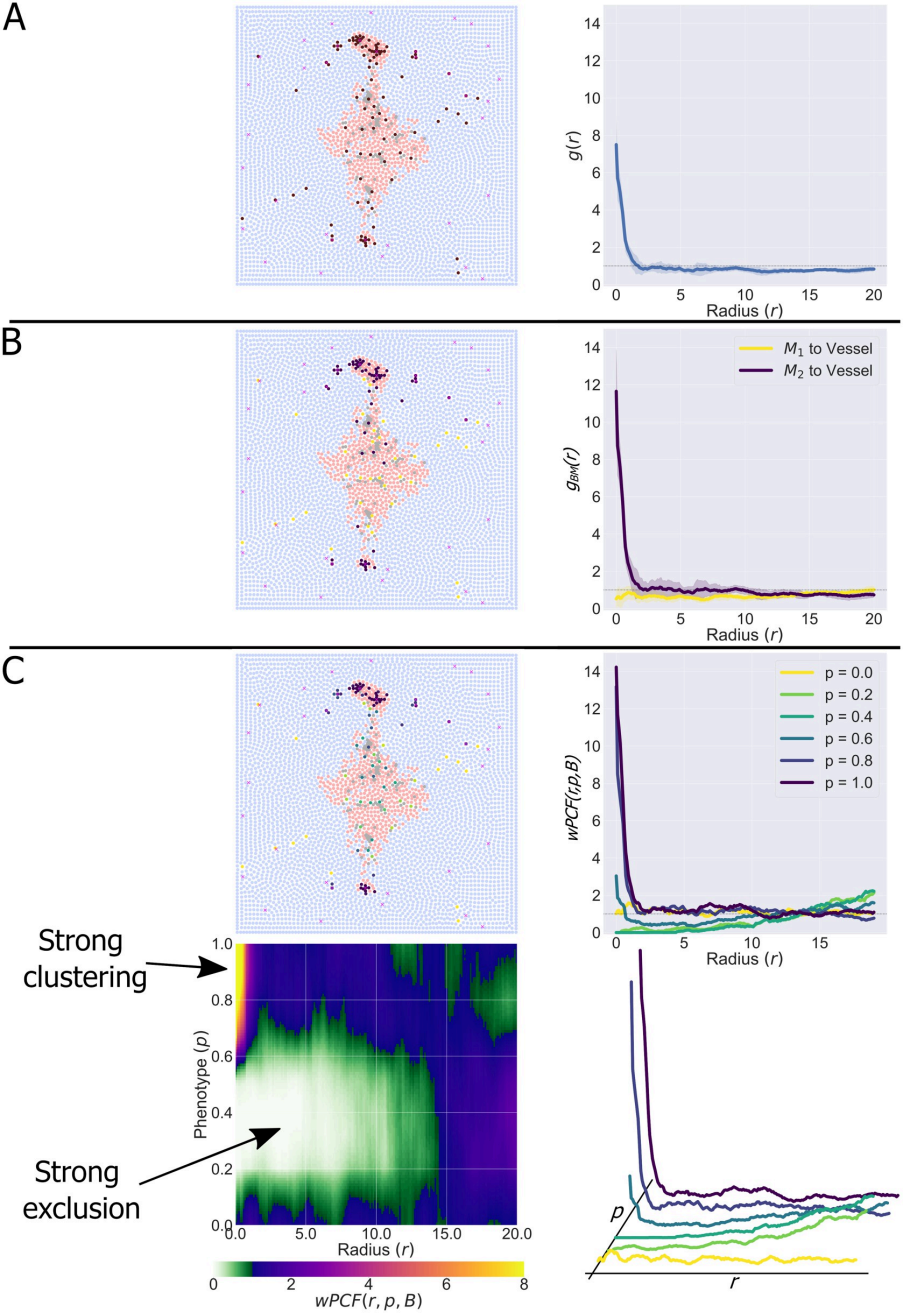
wPCF shows how macrophage phenotype and spatial distribution are related. A: Treating macrophages as a single population shows clustering between macrophages and tumour cells in the cross-PCF. B: Defining two populations of macrophages (M_1_, with *p* ≤ 0.5, or M_2_ with *p* > 0.5) shows differences in spatial localisation: M_1_ macrophages are randomly spread through the domain, while M_2_ macrophages are clustered around blood vessels. C: Using the full phenotype spectrum reveals three macrophage subpopulations, which are clearly visible in the wPCF. Macrophages with *p* ≈ 0 have no significant spatial relationship with blood vessels, macrophages with 0.6 ⪅ *p* have strong short range colocalisation with blood vessels, and macrophages with 0.1 ⪅ *p* ⪅ 0.6 are strongly excluded from blood vessels at distances up to 15 cell diameters. As well as *wPCF*(*r*, *p*, *B*) (bottom left), we present cross-sections of the wPCF (top and bottom right) which show that the wPCF has a similar interpretation as the cross-PCFs in A and B, while providing greater resolution in macrophage phenotype.

Fig 5 illustrates how resolving macrophage phenotype as a continuous variable enhances interpretation of the spatial patterns that macrophages adopt in solid tumours.

In Fig 5A, macrophage phenotype is not resolved. The cross-PCF between macrophages and blood vessels reveals strong short-range clustering. In Fig 5B the macrophages are partitioned into two subpopulations: without loss of generality, M_1_ macrophages have *p* ≤ 0.5 while M_2_ macrophages have *p* > 0.5. We calculate the cross-PCF between each macrophage subpopulation and blood vessels. The resulting cross-PCFs show that M_2_ macrophages are strongly clustered around blood vessels, while the M_1_ macrophages are not significantly associated with the blood vessels at any length scale.

In Fig 5C macrophage phenotype is viewed as a continuous variable and we compute the wPCF between the macrophages and the blood vessels (*wPCF*(*r*, *p*, *B*)). The wPCF identifies three distinct macrophage populations, rather than the two populations used in Fig 5B. The spatial positions of macrophages with *p* ≈ 0 and blood vessels are not strongly correlated, as for the M_1_ population in Fig 5A. Macrophages with 0.6 ⪅ *p* exhibit strong short range colocalisation with blood vessels, as for the M_2_ population in Fig 5B. The wPCF identifies a third population of macrophages with 0.1 ⪅ *p* ⪅ 0.6 which is strongly excluded from blood vessels at distances up to approximately 15 cell diameters. This distance corresponds to the approximate distance from blood vessels to the tumour core, suggesting that these macrophages are localised inside the tumour mass.

### wPCFs produce signatures that distinguish the 3 Es of immunoediting

**Fig 6:**
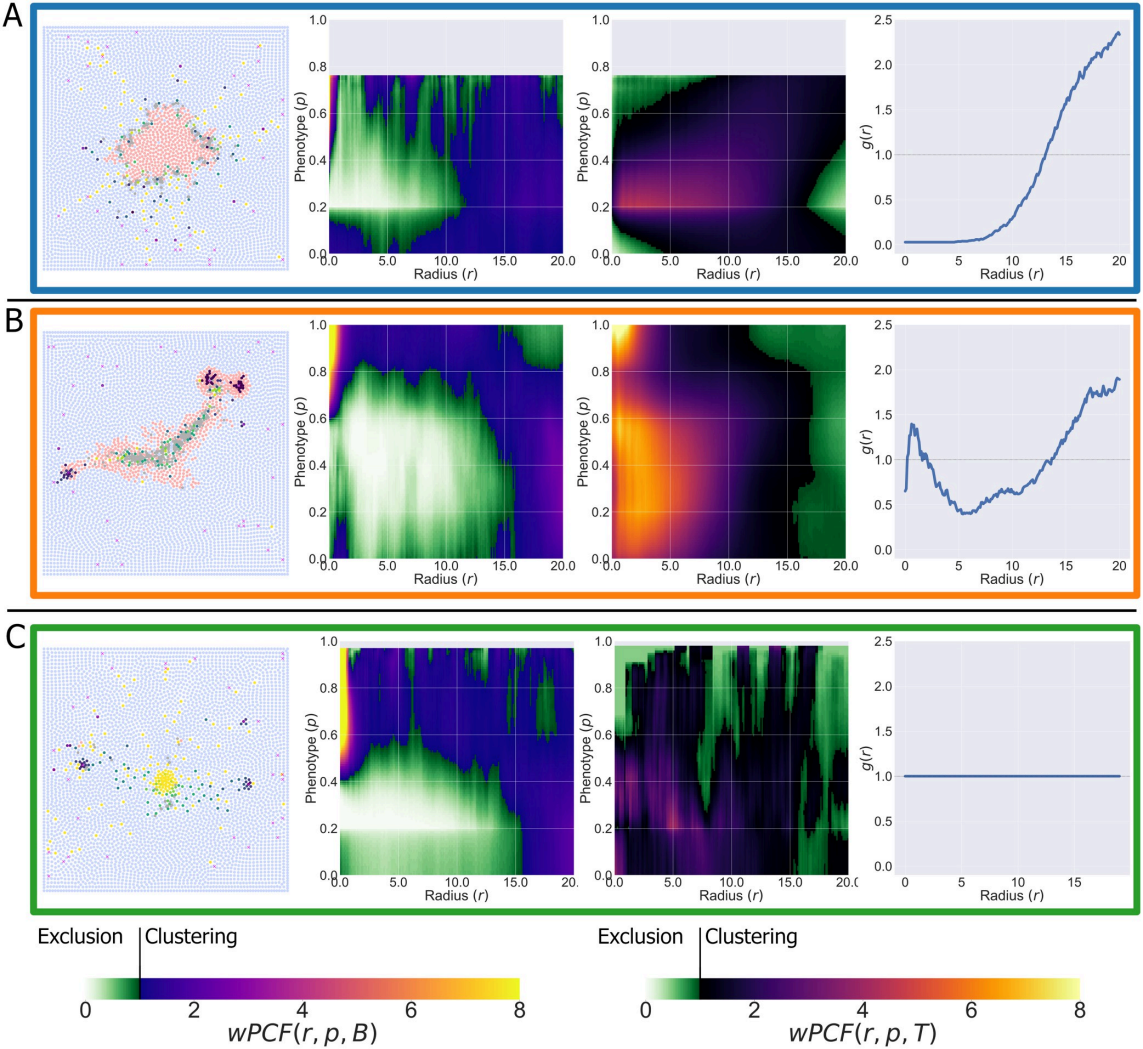
‘PCF signatures’ for Equilibrium, Escape and Elimination. We consider parameter combinations representing Equilibrium (A, $\chi_{c}^{m}=1,c_{1/2}=0.8$), Escape (B, $\chi_{c}^{m}=3.5,c_{1/2}=0.7$) and Elimination (C, $\chi_{c}^{m}=1.5,c_{1/2}=0.3$). For each, we show a representative simulation at *t* = 500. The wPCFs describing macrophage relationships with blood vessels and macrophage relationships with tumour cells are shown, alongside the cross-PCF describing blood vessel to tumour cell relationships (each averaged over 10 simulation repetitions).

In Fig 6 we analyse the spatial patterns generated by the ABM for different values of $\chi_{c}^{m}$ and *c*_1/2_. These patterns resemble Equilibrium (A, $\chi_{c}^{m}=1,c_{1/2}=0.8$), Escape (B, $\chi_{c}^{m}=3.5,c_{1/2}=0.7$) and Elimination (C, $\chi_{c}^{m}=1.5,c_{1/2}=0.3$). For each simulation, we compute *wPCF*(*r*, *p*, *B*), *wPCF*(*r*, *p*, *T*) and *g*_*BT*_(*r*), to characterise the pairwise spatial relationships between macrophages of different phenotypes, blood vessels, and tumour cells. We define the combination of these three statistics as a ‘PCF signature’ for our simulations.

Equilibrium simulations (Fig 6A) may not contain all macrophage phenotypes. Therefore, their wPCFs may be undefined for some values of *p* (in this case, for 0.78 ⪅ *p*.) There is a marked difference in the spatial localisation of macrophages with *p* ≈ 0 and those with *p* > 0.2, with mid-phenotype macrophages exhibiting short-range exclusion from blood vessels but not from tumour cells. In this case, macrophages which have not been exposed to TGF-*β* (*p* = 0) are restricted to the stroma while those with larger phenotype values cluster around the tumour mass, at distance from the blood vessels. The cross-PCF between blood vessels and tumour cells, *g*_*BT*_(*r*), indicates strong exclusion between blood vessels and tumour cells at distances of up to approximately 12.5 cell diameters, which is comparable to the exclusion distance between macrophages of intermediate phenotype and blood vessels.

For the Escape simulations (Fig 6B), macrophages with *p* = 1 cluster (*r* ≈ 0) with blood vessels and tumour cells. With the peak in *g*_*BT*_(*r*) near *r* = 0, this indicates the formation of perivascular niches containing tumour cells, blood vessels and M_2_ macrophages, as reported by Arwert *et al*. [33]. Macrophages with an intermediate phenotype are strongly excluded from blood vessels at radii up to 15 cell diameters and strongly associated with tumour cells at radii up to approximately 10 cell diameters. Taken together with the exclusion of tumour cells from blood vessels indicated by *g*_*BT*_(*r*) for 2.5 ⪅ *r* ⪅ 12.5, this is characteristic of a central tumour mass populated with transitioning macrophages and the formation of perivascular niches.

Finally, for Elimination simulations (Fig 6C), there are no strong correlations between tumour cells and macrophages and *wPCF*(*r*, *p*, *T*) is extremely noisy (because there are very few tumour cells). Similarly, *g*_*BT*_(*r*) ≈ 1, since most simulations with this parameter set have no tumour cells. *wPCF*(*r*, *p*, *B*) is similar to that shown in Fig 6B, indicating that the macrophage distribution for Elimination is similar to that for Escape (M_2_ macrophages cluster around blood vessels, and transitioning macrophages localise in the domain centre, at distance from the vasculature). This further suggests that the time courses for Elimination and Escape simulations may be similar at early times.

### Dimension reduction via PCA permits quantitative comparison of PCF signatures

**Fig 7:**
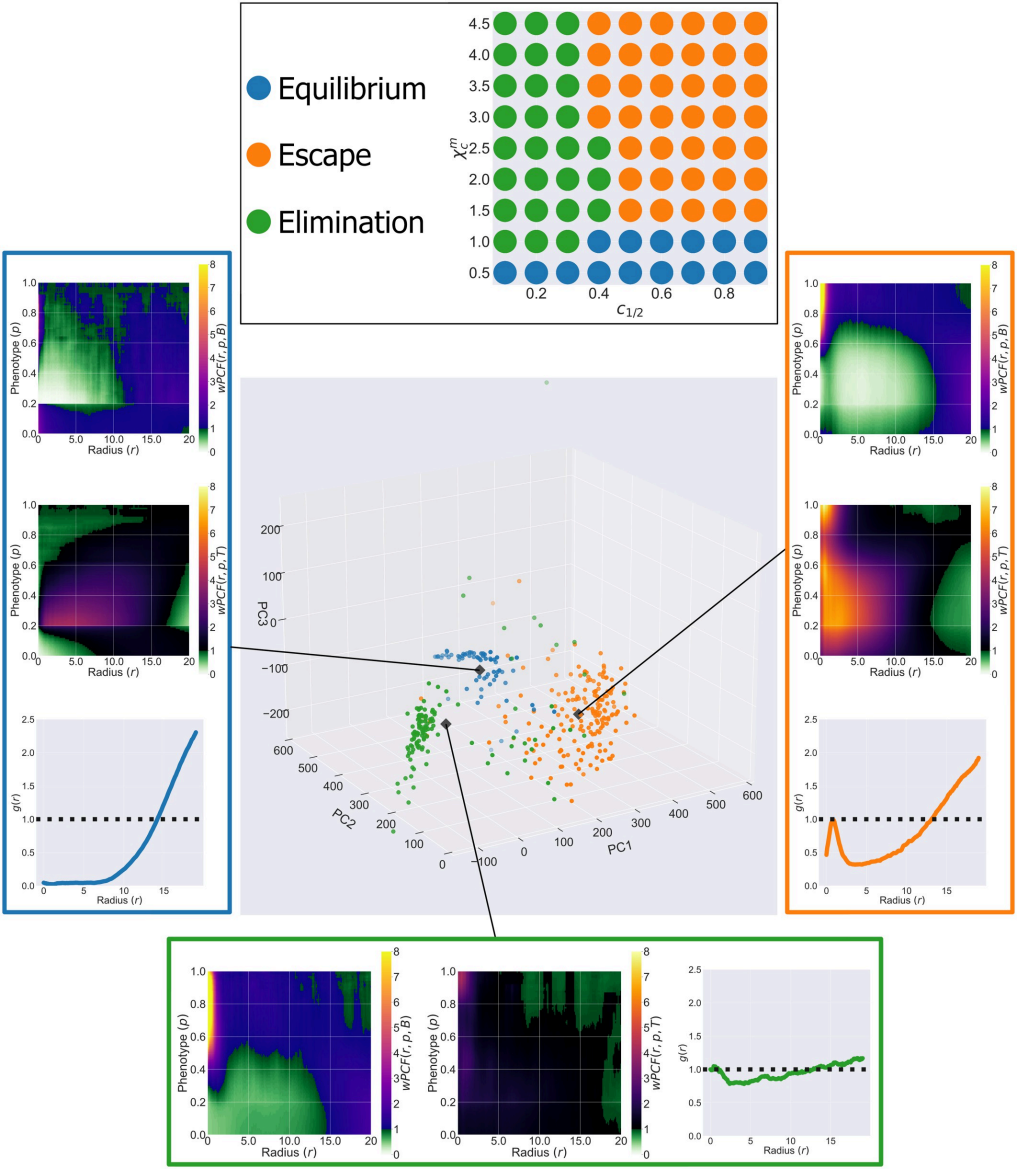
Projecting PCF signatures onto their first three principle components resolves the 3 Es of immunoediting. Top: labels assigned to each parameter combination at *t* = 500. Labels are manually assigned based on the predominant behaviour observed across all realisations of that parameter set. Main: Projection of the vectorised PCF signatures from outputs at *t* = 500 of 371 ABM simulations onto their first three principal components. Simulations cluster according to their label (manually defined as Escape, Elimination or Equilibrium as per the inset above). The top 100 principal components for the centroid of each cluster have been converted back into wPCF and cross-PCF signatures, and the corresponding *wPCF*(*r*, *p*, *B*), *wPCF*(*r*, *p*, *T*) and *g*_*BT*_(*r*) to each are shown. Each inset has the same interpretation as the PCF signatures in Fig 6, showing that conversion between PCF signatures and PCA-space is straightforward.

The parameter sweep described in Fig 4 contained 737 individual simulations, with 7–10 stochastic realisations conducted for each parameter combination (limited by availability of HPC resources). Each image was manually classified as Equilibrium, Escape or Elimination according to the most prominent behaviour displayed (see inset of Fig 7 for parameter combinations and labels). We allocated 371 of these images into a training dataset (simulations with ‘iteration number’ ∈ [0, 4]) and the remaining 366 into a testing dataset (simulations with ‘iteration number’ ∈ [5, 9]). For each image, we computed *wPCF*(*r*, *p*, *B*), *wPCF*(*r*, *p*, *T*) and *g*_*BT*_(*r*) to form the PCF signature described in the previous section. We then vectorised and concatenated the three spatial statistics, to form a high-dimensional vector (38,773 entries). This process is described more fully in S6 Appendix.

We then applied dimensionality reduction to the training dataset. In Fig 7, we use principal component analysis (PCA) to project these high-dimensional vectors onto their first three principal components (the first principal component lies in the direction that maximises the variance in the data; each successive principal component maximizes the remaining variance in the data and is orthogonal to all previous principal components). Projecting the data onto the first 3 principal components shows that the synthetic images cluster according to their labels, suggesting that *wPCF*(*r*, *p*, *B*), *wPCF*(*r*, *p*, *T*) and *g*_*BT*_(*r*) capture sufficient information to distinguish between the three qualitative behaviours that the ABM exhibits (i.e., the three Es of immunoediting).

The wPCFs and PCFs associated with the centroids of each cluster are presented in the inset in Fig 7, and are obtained by summing the first 100 principal components that define each centroid. These PCF signatures are consistent with those presented in Fig 6, and suggest that the first three principal components associated with the simulation output from the ABM at *t* = 500 could be used to classify it as Escape, Elimination or Equilibrium.

The 371 data points shown in Fig 7 were then used as training data for a support vector machine (SVM) with a radial basis function kernel (default implementation within python’s scikit-learn Support Vector Classification implementation), based on the first 100 principal components of the PCF signatures. We used the SVM to predict the labels of a testing set of the 366 simulations in the test dataset, and obtained 91.5% accuracy.

We applied the classifier to simulation outputs from a second parameter sweep, this time randomly choosing values of the same two parameters as previously ($\chi_{c}^{m}$ and *c*_1/2_) alongside randomly choosing four additional parameters ($\chi_{\xi }^{m}$, $\chi_{\epsilon }^{T}$, *c*_1/2_ and *g*_crit_) in order to generate a wider potential variety of simulation outcomes (see S7 Appendix for examples). This produced 431 additional images, which were manually labelled as Equilibrium, Escape or Elimination at *t* = 500. Table 1 shows the performance of the classifier at predicting the labels of these simulations. The classifier identifies Escape simulations with extremely high accuracy, correctly identifying 97.2% of simulations in which tumour intravasation and metastasis is present. The overall classification accuracy, across all three possible outcomes, is 82.1%.

**Table 1:** Classification performance for 100 principal components. Classifier accuracy (number of classifications and percentage of true classifications assigned to that class) for SVMs trained on the first 100 principal components, based on 431 manually labelled simulations at *t* = 500 with parameters randomly sampled within a 6-parameter space. Bold fields show correct classifications.

		Predicted classification		
		Equilibrium	Escape	Elimination
True classification	Equilibrium	**78 (65%)**	26 (22%)	16 (13%)
	Escape	0 (0%)	**104 (97%)**	3 (3%)
	Elimination	18 (9%)	14 (7%)	**172 (84%)**

### ABM simulations may transition between the three Es of immunoediting over time

We have shown that ABM data from a single timepoint can be classified as Equilibrium, Escape or Elimination based on their PCF signatures. Since the ABM simulations are dynamic, we can use the methods used to create Fig 7 to investigate how the qualitative behaviour of an ABM simulation changes over time.

**Fig 8:**
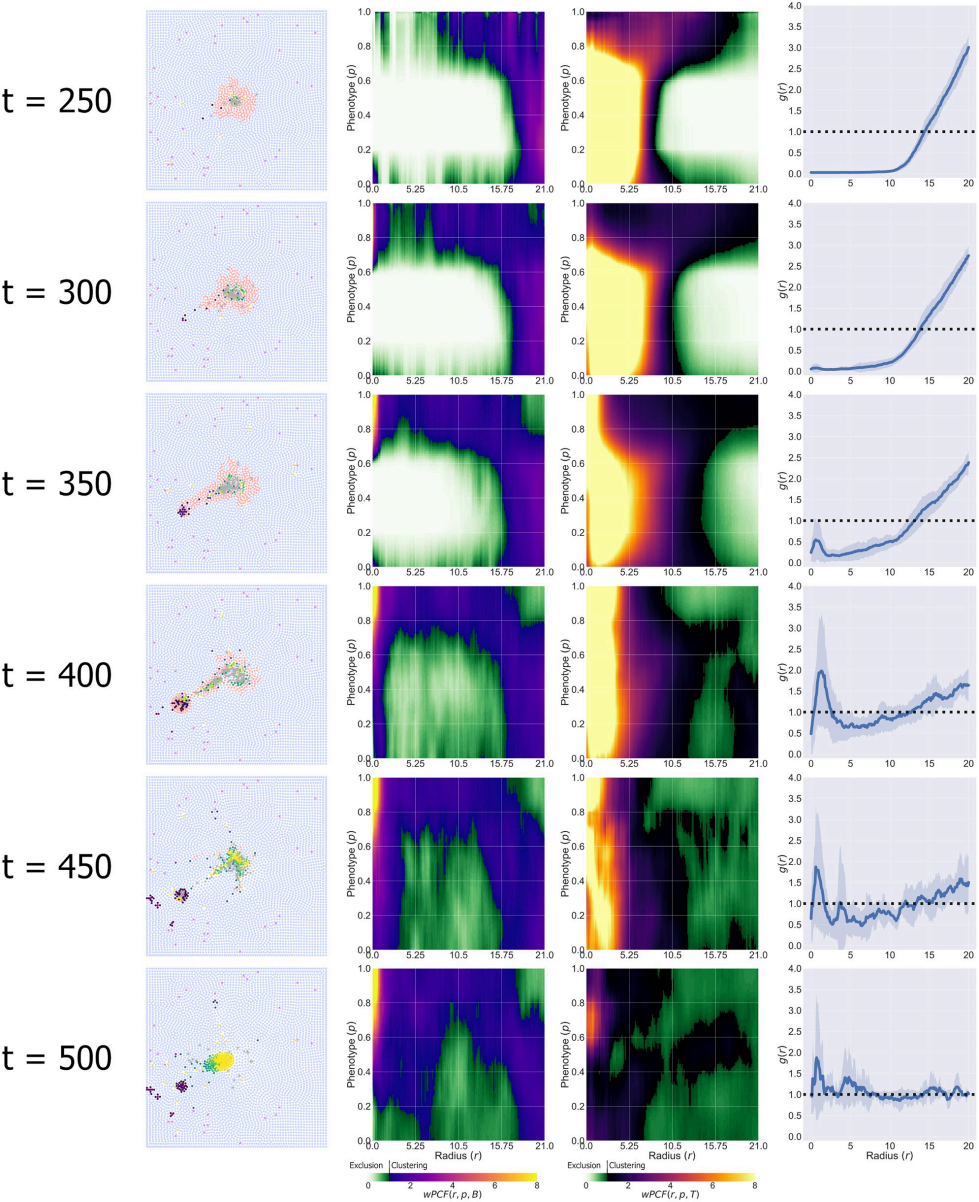
Dynamic evolution of an ABM simulation. Time series showing the evolution of an ABM simulation generated with $\chi_{c}^{m}=4.5$ and *c*_1/2_ = 0.3, and all other parameter values fixed at the default values listed in S1 Appendix. At different timepoints, the simulation exhibits behaviours which are consistent with Equilibrium, Escape and Elimination. The corresponding PCF signatures show how the ABM progresses through these stages.

The results presented in Fig 8 derive from an ABM simulation with $\chi_{c}^{m}=4.5$ and *c*_1/2_ = 0.3, which we classify as ‘Elimination’ based on its output at *t* = 500. The time series in Fig 8 show how, as the tumour develops, the simulation transitions from ‘Equilibrium’ (compact mass, *t* = 250) through to ‘Escape’ (*t* = 350, 400) and, ultimately, to ‘Elimination’.

**Fig 9:**
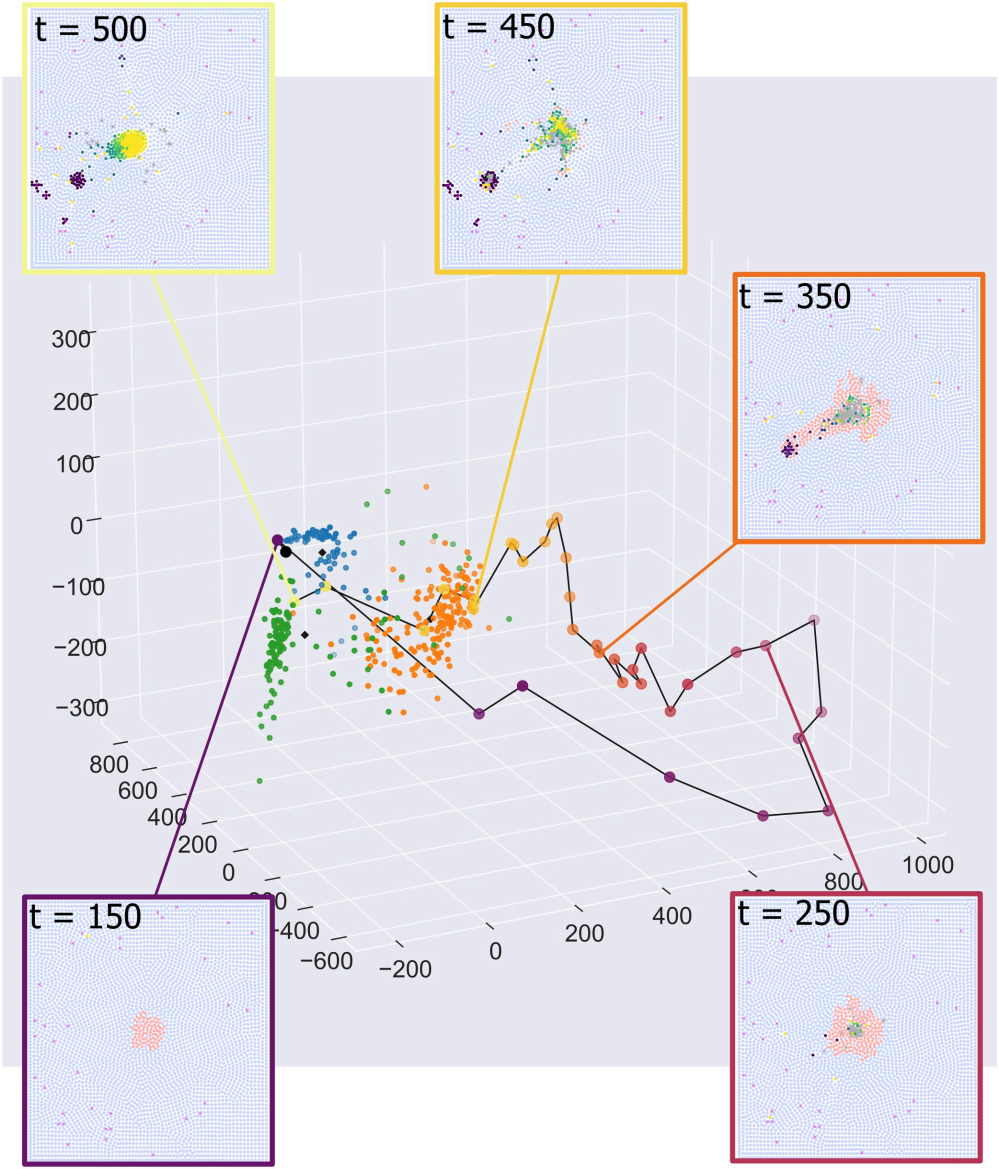
Dynamic evolution of an ABM simulation through PCA space. A single simulation can exhibit Equilibrium (*t* = 150), Escape (*t* = 350, 450) or Elimination (*t* = 500) at different times. These changes are captured by the movement of the PCF signature through PCA space.

We calculate PCF signatures for this ABM simulation every 10 hours, and project them onto the first three principal components, using the process in Fig 7. The resulting trajectory is depicted in Fig 9, with points coloured according to their time and the insets showing the corresponding synthetic images.

At early times, the images localise within the Equilibrium cluster (blue cluster; *t* = 150). Once macrophages start to appear in the images, the trajectory moves away from the three clusters, due to noise in the wPCFs caused by the small number of macrophages relative to the *t* = 500 simulations which define the clusters (*t* = 250). As the number of macrophages increases, the trajectory moves closer to the Escape cluster (orange cluster; *t* = 350 and *t* = 450). Finally, as the tumour cells are killed and removed from the simulation, the trajectory moves into the Elimination cluster (green cluster; *t* = 500).

This study highlights two related challenges. First, spatial data from a single timepoint may not be predictive of past or future behaviour: an ABM may exhibit multiple behaviours at different timepoints. Second, for this simulation, some tumour cells successfully migrate to neighbouring blood vessels prior to the tumour’s elimination. In practice, these tumour cells could enter the vasculature and spread to other parts of the body. Given information about the tumour’s spatial composition at *t* = 500 only, we will classify this image as Elimination without identifying the Escape behaviour present at earlier times.

## Discussion

In this paper we have introduced a new spatial statistic, the wPCF, which extends the cross-PCF to point clouds labelled with a mixture of categorical and continuous labels. We demonstrated its utility by applying it to synthetic data generated from a new ABM that simulates macrophage interactions with a tumour growing in a 2D vascular tissue. Blood vessels and tumour cells are categorically labelled, while macrophages have a continuous phenotypic label. The wPCF reveals spatial correlations between the different cell types.

The ABM focusses on the impact that phenotypic heterogeneity in the macrophage population has on the tumour’s patterns of growth. By varying parameters that control macrophage sensitivity to environmental cues, we used the ABM to generate a range of synthetic data that spans the three Es of immunoediting (Equilibrium, Escape or Elimination). We showed that wPCFs and cross-PCFs can be combined to produce a high dimensional ‘PCF signature’ which characterises the relative locations of macrophages, tumour cells and blood vessels. Our results suggest that, given suitable training data, the PCF signature could be used to automate the classification of images or point patterns into different clusters, by using PCA to project the PCF signature onto a lower dimensional space.

One advantage of testing the wPCF on synthetic data from an ABM is its ability to generate time-series data. Our analysis of dynamic output from an ABM simulation showed how the PCF signature may vary over time. A given simulation may transition between different states, suggesting that caution is needed when using a single time point to make predictions about a tumour’s future growth and response to treatment. This study also highlights one of the benefits of developing mathematical models of a biological system: the ABM can be used to investigate questions that would be challenging to address with existing experimental techniques. For example, it is not currently feasible to apply multiplex imaging to the same tissue at multiple timepoints.

There are many directions for extending and improving the ABM. For example, including tumour cell intravasation would prevent situations in which an Escape signature can progress to Elimination at a later timepoint, as in Fig 8. In practice, tumour cells would have entered the vasculature and may have established metastases. In future work, we will also explore ABM extensions which incorporate therapies, such as radiotherapy or immunotherapy, and investigate whether interactions between different cell types are predictive of response (see, e.g., [57–59]). We will consider whether the spatial distributions of cells at different timepoints must be accounted for when making such predictions. We could further extend the ABM to account for multiple immune cell subtypes, such as T cells, or stromal cells such as fibroblasts [60].

The robustness of the wPCF should be tested through application to other types of ABMs that simulate tumour growth and generate similar outputs. In addition to the Chaste framework used here [51–53], other candidate ABM frameworks include PhysiCell [61], HAL (Hybrid Automata Library) [62], and CompuCell3D [63, 64].

In this paper we used the wPCF and cross-PCF to describe and classify synthetic data generated by an ABM. However, the wPCF has application to a wider range of scientific fields. In particular, the wPCF can account for continuous variation in expression levels of cellular markers which characterise multiplex imaging modalities, such as multiplex immunohistochemistry or Imaging Mass Cytometry. Equally, the wPCF could be used to describe any structured cell population, for example continuous labels describing the stemness or differentiation status of cancer cells, the exhaustion level of T-cells, or the oxygen concentration experienced by cells within a tissue sample. Beyond biology, the wPCF could be used to analyse data from other applications in which PCFs have proven useful, including astronomy [19, 20] and ecology [16–18]. We note that, as with the PCF and cross-PCF, it is straightforward to generalise the wPCF for data in 1D or 3D, making it applicable outside of purely 2-dimensional imaging data. The wPCF can be extended to compare the spatial distributions of point patterns with multiple continuous labels. In Fig 1 expression levels of CD68, CD163, and CD204 were used to define distinct macrophage subtypes; each marker could represent a separate continuous structure label. We show how the wPCF can be used to describe the spatial distribution of points with two continuous labels in S5 Appendix.

Future work will involve applying the wPCF to multiplex imaging data, in order to validate its use in biological and clinical settings. Applying such statistics to medical images would enable their high-throughput, automated quantification and comparison in a manner that goes beyond expert visual inspection and is more interpretable than AI approaches [65, 66]. We note also that while in this paper we focus on correlation functions, alternative metrics, including topological data analysis, can describe spatial features such as immune deserts that exist in noisy data [30], or changes in tumour and vascular architecture in response to radiotherapy [67]. Multiple spatial statistics can be combined to obtain more detailed descriptions of 2D data [68], or new statistics can be derived from networks of cell contact [69] or observations of immune cell locations [70, 71].

In this paper we presented a proof-of-concept SVM classifier to show how multiple statistics can be combined to classify data, with PCA acting as a dimension reduction technique which permits a classifier to be trained on high dimensional statistics without sacrificing their interpretability (as is generally required for AI approaches). This method has the potential to bridge the gap between classifiers applied to summary statistics, which are flexible and fast to train but generally do not include spatial data, and classifiers applied directly to images, which account for spatial features of images but are difficult to interpret, and require large training datasets and intensive computation. While the classifier described here works, there is considerable scope for optimisation in relation to i) the choice of statistics, ii) the choice of classification method, and iii) the choice of dimension reduction tool. In future work, we will explore alternative choices at each of these stages, in order to improve our pipeline for classification of point clouds based on their spatial structure. We will also use a wider range of techniques, such as topological data analysis, to characterise the outputs from different simulations and timepoints.

This paper demonstrates an exciting proof-of-concept: statistics which describe different aspects of cell localisation can be combined to classify, describe and analyse synthetic and biological point clouds.

## Supporting information

S1 Appendix Model description. (PDF)

S2 Appendix Tumour progression in the presence and absence of macrophages. (PDF)

S3 Appendix Derivation of PCFs and cross-PCFs from wPCF. (PDF)

S4 Appendix Comparison of different weighting functions. (PDF)

S5 Appendix wPCF for comparing two continuous labels. (PDF)

S6 Appendix Vectorisation and support vector machine classification. (PDF)

S7 Appendix Variation in simulation behaviour for a wider range of parameters. (PDF)
