# Supplementary material for: Quantification of spatial and phenotypic heterogeneity in an agent-based model of tumour-macrophage interactions
Source: PLoS Comput Biol. 2023 Mar 27;19(3):e1010994. doi: 10.1371/journal.pcbi.1010994 (PMC10079237; doi:10.1371/journal.pcbi.1010994)
Supplement: S1 Appendix — (PDF) [file pcbi.1010994.s001.pdf]

S1 Appendix: Model Description

Our ABM extends an existing ABM that describes infiltration of microbeads [1] and macrophages [2] into tumour spheroids growing *in vitro*. Here, we consider an *in vivo* scenario, where tumour cells are embedded within a tissue containing stromal cells and oxygen is supplied by blood vessels. We use an overlapping-spheres model, representing each cell as a point whose movement and position are determined by balancing the forces that act on it. We distinguish four types of agent: **macrophages**, **tumour cells**, **stromal cells** and **necrotic cells**. We also consider five diffusible species: **oxygen** ($\omega(\mathbf{x},t)$), **colony stimulating factor-1** (CSF-1, $c(\mathbf{x},t)$), **transforming growth factor-**β (TGF-β, $g(\mathbf{x},t)$), **epidermal growth factor** (EGF, $\epsilon(\mathbf{x},t)$), and **C-X-C motif chemokine 12** (CXCL12, $\xi(\mathbf{x},t)$). Their dynamics are defined by reaction-diffusion equations (RDEs). Following [1,2], we use the open source Chaste (Cancer, Heart and Soft Tissue Environment) modelling environment to generate simulation [3–5].

Diffusible species

The distribution of the diffusible species is modelled using RDEs, with each equation solved numerically on a triangular finite element mesh that spans the domain. We consider a square domain Ω, with height and width equal to 50 cell diameters (approximately 1 mm in dimensional units). In this Section we describe the role of each diffusible species, the factors that govern their rates of production and depletion, and the RDEs that define their evolution. Fig 2 in the main text summarises how the diffusible species interact with tumour cells and macrophages. Since we assume oxygen and CXCL12 are produced from the (static) blood vessels, we make a quasi-steady state assumption for their distribution. By contrast, changes in the distributions of EGF, TGF-β and CSF-1 are assumed to happen on the slower timescale associated with tumour cell and macrophage movement. Therefore we do not make the quasi-steady assumption when calculating these distributions.

Oxygen (ω) Oxygen is supplied by blood vessels which are represented by static point sources, and is consumed by tumour cells and stromal cells as it diffuses through the domain. We assume that the oxygen concentration at each blood vessel is constant, ω_0.

Since the timescale of diffusion for an oxygen molecule is faster than the timesteps used in our model to describe cell movement, we assume that the distribution of oxygen can be approximated using a steady state solution [1,2]. For simplicity, we rescale the oxygen concentrations with a factor of ω_0, so that $\omega = 1$ at each blood vessel. The governing equation is then given by

$$0 = D_\omega \nabla^2 \omega - \kappa_\omega \omega \sum_i \delta(\mathbf{x} - \mathbf{x}_i) \tag{1}$$

for $\mathbf{x} \in \Omega$, where $\mathbf{x}_i$ is the location of stromal or tumour cell i, the parameter D_ω is the assumed constant diffusion coefficient of oxygen, κ_ω is the oxygen consumption rate and δ is the delta function ($\delta(\mathbf{x}) = 1$ when $\mathbf{x} = 0$, $\delta(\mathbf{x}) = 0$ otherwise). We impose Neumann boundary conditions ($\frac{\partial \omega}{\partial \mathbf{x}} = 0$) on domain boundaries, and assume that initially $\omega(\mathbf{x}, t = 0) = 1$ for all $\mathbf{x}$ that do not represent a blood vessel.

CXCL12 (C-X-C motif chemokine 12, ξ) CXCL12 is produced by perivascular cancer-associated fibroblasts (CAFs) and acts as a diffusible chemoattractant for M_2 macrophages [6]. We suppose that CAFs localise close to blood vessels, and, hence, make the simplifying assumption that blood vessels act as constant sources of CXCL12. We therefore assume that the concentration of CXCL12, ξ, is maintained at a fixed

value $\xi = \xi_0$ at all blood vessels. We assume that CXCL12 decays naturally at a constant rate, λ_ξ. The distribution of CXCL12 in the domain is then given by

$$0 = D_\xi \nabla^2 \xi - \lambda_\xi \tag{2}$$

where $D_\xi$is the diffusion coefficient for CXCL12. We impose Neumann boundary conditions ($\frac{\partial \xi}{\partial \mathbf{x}} = 0$) on domain boundaries, and assume that initially $\xi(\mathbf{x}, t = 0) = 0$ for all $\mathbf{x}$ that do not represent a blood vessel.

CSF-1 (Colony stimulating factor-1, c) The macrophage chemoattractant CSF-1 is produced by tumour cells. It acts with EGF as part of a paracrine loop [7, 8] and stimulates macrophage extravasation [6]. We assume that CSF-1 is produced at a constant rate, κ_c, by each tumour cell, and decays at a constant rate, λ_c. The distribution of CSF-1 is therefore described by the equation

$$\frac{\partial c}{\partial t} = D_c \nabla^2 c - \lambda_c c + \kappa_c \sum_i \delta(\mathbf{x} - \mathbf{x}_i) \tag{3}$$

where D_c is the diffusion coefficient of CSF-1 and the sum runs over all tumour cells i. We impose Neumann boundary conditions ($\frac{\partial c}{\partial \mathbf{x}} = 0$) on domain boundaries, and assume that initially $c(\mathbf{x}, t = 0) = 0$ for all $\mathbf{x}$.

TGF-β (Transforming growth factor-β, g) TGF-β causes macrophages to alter their phenotype, and is produced by tumour cells. We suppose that it decays at a constant rate, λ_g, and is produced by tumour cells at a constant rate, κ_g. Combining these effects, we arrive at the following RDE for $g(\mathbf{x}, t)$:

$$\frac{\partial g}{\partial t} = D_g \nabla^2 g - \lambda_g g + \kappa_g \sum_i \delta(\mathbf{x} - \mathbf{x}_i) \tag{4}$$

where D_g is the diffusion coefficient of TGF-β and the sum is over all tumour cells, i. As for CSF-1, we impose Neumann boundary conditions ($\frac{\partial g}{\partial \mathbf{x}} = 0$ on domain boundaries), and assume that $g(\mathbf{x}, t = 0) = 0 \ \forall \mathbf{x} \in \Omega$.

EGF (Epidermal growth factor, ϵ) EGF is a diffusible chemoattractant for tumour cells that is produced by tumour associated macrophages. We assume that EGF is produced by macrophages at a rate that is linearly dependent on their phenotype p, so that macrophages with an extreme M_1 phenotype ($p = 0$) produce no EGF and macrophages with an extreme M_2 phenotype ($p = 1$) produce κ_ϵ. We assume that EGF naturally decays at a constant rate, λ_ϵ. Combining these effects, we obtain the following RDE for EGF, $\epsilon(\mathbf{x}, t)$:

$$\frac{\partial \epsilon}{\partial t} = D_\epsilon \nabla^2 \epsilon - \lambda_\epsilon \epsilon + \kappa_\epsilon \sum_i p_i \delta(\mathbf{x} - \mathbf{x}_i) \tag{5}$$

where D_ϵ is the constant diffusion coefficient of EGF, κ_ϵ is the maximum rate of production of EGF, the sum is over all macrophages i, and $p_i \in [0, 1]$ is the phenotype of macrophage i and is defined below. As for CSF-1 and TGF-β, we impose Neumann boundary conditions ($\frac{\partial \epsilon}{\partial \mathbf{x}} = 0$ on the boundaries of the domain Ω), and assume that $\epsilon(\mathbf{x}, t = 0) = 0 \ \forall \mathbf{x} \in \Omega$.

Agents

Our model distinguishes four types of agents: **macrophages**, **tumour cells**, **stromal cells** and **necrotic cells**. A fixed number of static **blood vessels** are also distributed throughout the domain. The location of each agent is represented by its cell centre.

We use Newton's second law to determine the equations of motion for macrophages, tumour cells, stromal cells and necrotic cells. Neglecting inertial effects in the overdamped limit, the force balance for cell i can be written as:

$$\nu \frac{d\mathbf{x}_i}{dt} = \mathbf{F}_i, \tag{6}$$

where ν is the drag coefficient (assuming that the drag on a cell is proportional to its velocity), and $\mathbf{F}_i$ denotes the net force acting on cell i. The forces that act on each cell type are indicated in Fig 2 in the main text.

All cells are subject to mechanical forces due to cell-cell interactions (incorporating repulsion due to volume exclusion and attraction due to intercellular adhesion). Here we use the overlapping spheres approach [9, 10], assuming that two cells interact if the distance between their cell centres is less than a fixed distance R_{int}. Specifically, for cells at locations $\mathbf{x}_i$ and $\mathbf{x}_j$, if $|\mathbf{x}_i - \mathbf{x}_j| < R_{\text{int}}$ then the interaction force between cells i and j is parallel to the vector $(\mathbf{x_i} - \mathbf{x_j})$ connecting their centres. The resting spring length between the cell centres, $s_{i,j}$, is the sum of the equilibrium spring lengths associated with each cell ($s_{i,j} = s_i + s_j$). For most cells i, the resting spring length is equal to the approximate radius of a cell ($s_i = R_{\text{Cell}} \equiv 0.5$ in non-dimensional units). For newly divided and necrotic cells, s_i changes over time to account for cell growth and shrinkage (see below).

The net mechanical force acting on cell i is the sum of all interaction forces due to cells within its interaction radius:

$$\mathbf{F}_i^m = \sum_{\{j \,:\, |\mathbf{x}_i - \mathbf{x}_j| \le R_{\text{int}}\}} \mathbf{F}_{i,j}^m. \tag{7}$$

where $\mathbf{F}_{i,j}^m$ is the mechanical force between cells i and j. This force always points in the direction of the vector connecting the cell centres and has magnitude:

$$|\mathbf{F}_{i,j}^m| = \begin{cases} \mu s_{i,j} \log\left(1 + \frac{x}{s_{i,j}}\right) \text{ if } x < 0 \text{ (Repulsive)} \\ \mu x s_{i,j} \exp\left(-\alpha \frac{x}{s_{i,j}}\right) \text{ if } x \ge 0 \text{ (Adhesive)} \end{cases} \tag{8}$$

where $x = |\mathbf{x}_i - \mathbf{x}_j| - s_{i,j}$ is the overlap between cells i and j, μ is a parameter describing the spring stiffness and α determines the strength of intercellular adhesion between neighbouring cells.

We normalise lengths with the lengthscale R_{Cell}, assuming that 1 cell diameter $= 2R_{\text{Cell}} = 20\mu$m. Following cell division, the radius of the daughter cells is set to $s_i = \frac{R_{\text{Cell}}}{2}$ and increases linearly over one hour until $s_i = R_{\text{Cell}}$. For necrotic cells, s_i decreases linearly to zero over $\bar{\tau}_i$ hours (see below), and then the cell is removed from the simulation. The associated spring stiffness μ of springs attached to a necrotic cell is reduced linearly at the same rate.

Macrophages

Extravasation Macrophages extravasate from blood vessels in response to local levels of CSF-1. At each timestep, the probability that a macrophage extravasating in a one hour time period is P_{ex}, where:

$$P_{\text{ex}} = P^{\star} \times \frac{c}{c + c_{1/2}} \tag{9}$$

where the parameter $P^{\star}$ controls the maximum possible probability of macrophage extravasation per hour from each vessel, and $c_{1/2}$ is the concentration of CSF-1 at which this probability is half-maximal.

Phenotype The diffusible species included in our model bind to receptors on the outer membranes of macrophages and tumour cells and regulate behaviours including chemotaxis and the production of other species. While we do not explicitly model surface receptors, the receptor CXCR4 (C-X-C motif chemokine receptor type 4) plays an important role in this system: when exposed to TGF-β, macrophages increase expression of CXCR4 and become sensitive to gradients of CXCL12 [6]. We model this by associating each macrophage with a phenotype, $p \in [0, 1]$, which determines the extent to which it exhibits M_1- or M_2 behaviour. When a macrophage first enters the spatial domain, it has phenotype $p = 0$. Exposure to TGF-β causes its phenotype to increase irreversibly. The rate of change of the phenotype of macrophage i at location $\mathbf{x}_i$ is:

$$\frac{dp}{dt} = \mathcal{H}(g(\mathbf{x}_i, t) - g_{\text{crit}}) \times \mathcal{H}(1 - p) \times \Delta p \tag{10}$$

where $g(\mathbf{x}_i, t)$ is the concentration of TGF-β at $\mathbf{x}_i$ at time t, $\mathcal{H}(x)$ is the Heaviside function ($\mathcal{H}(x) = 0$ if $x < 0$, $\mathcal{H}(x) = 1$ otherwise), the non-negative parameter Δp determines the rate at which phenotype changes, and g_{crit} is a critical TGF-β threshold above which macrophage phenotype increases. When $p = 1$ the macrophage has a fully M_2 phenotype, and p no longer changes.

Phenotype affects the following macrophage behaviours:

- the rate at which they produce EGF;
- the rate at which they kill tumour cells;
- their chemotactic sensitivity to spatial gradients of CSF-1 and CXCL12.

Equation (5) describes how the rate of EGF production depends on p; we now describe how cell killing and phenotype-dependent chemotaxis are implemented.

Cell killing Macrophage phenotype determines the probability that a macrophage kills a tumour cell on a given timestep. The probability that macrophage i of phenotype p_i kills a tumour cell in one hour is

$$P_{\varphi} = \begin{cases} P_{\varphi}^{\star} \times (1 - \frac{p_i^{10}}{p_i^{10} + 0.5^{10}}) & \text{for } t_{\varphi} \geq t_{\text{cool}} \\ 0 & \text{otherwise} \end{cases} \tag{11}$$

where $P_{\varphi}^{\star}$ is the maximum probability that a macrophage kills a tumour cell in one hour. t_{φ} is the time since the macrophage last killed a tumour cell, and the parameter t_{cool} defines a 'cooldown' period during which the macrophage cannot kill another tumour cell. On each timestep the probability of each macrophage attempting cell killing is evaluated. If a macrophage attempts killing, any cells within distance 1 of the macrophage are identified and, if there is at least one tumour cell, one of the tumour cells is selected at random to be killed. The tumour cell is labelled as necrotic, and t_{φ} is reset to 0 for that macrophage.

Force laws for macrophages In addition to mechanical forces, macrophages are subject to forces which account for random movement and chemotactic forces due to spatial gradients of CXCL12 and CSF-1. We account for random movement via the force $\mathbf{F}_i^r = (F_x^r, F_y^r)$ where

$$\mathbf{F}_i^r = \sqrt{2Ddt}\,\boldsymbol{n}, \tag{12}$$

where the coefficient D describes the strength of the random force and $\boldsymbol{n} = (n_x, n_y)$, where n_x and n_y are random variables drawn from a standard normal distribution.

The net force due to chemotaxis acting on macrophage i depends on its phenotype, p_i, as follows:

$$\mathbf{F}_i^{\chi} = \chi_c^m (1 - p_i) \frac{\nabla c}{|\nabla c|} + \chi_\xi^m p_i \frac{\nabla \xi}{|\nabla \xi|} \tag{13}$$

where the parameters χ_c^m and χ_ξ^m control macrophage sensitivity to spatial gradients of CSF-1 and CXCL12 respectively. We suppose that macrophage sensitivity to CSF-1 and CXCL12 gradients scales linearly with phenotype.

The net force acting on macrophage i in Equation (6) is:

$$\mathbf{F}_i = \mathbf{F}_i^m + \mathbf{F}_i^r + \mathbf{F}_i^{\chi}. \tag{14}$$

Tumour cells

Cell cycle and proliferation Each tumour cell has an internal cell cycle which advances at a rate that depends on the local oxygen concentration and two oxygen thresholds, $\omega_{\mathrm{H}}^{\mathrm{tum}} < 1$ and $\omega_{\mathrm{N}}^{\mathrm{tum}} \leq \omega_{\mathrm{H}}^{\mathrm{tum}}$. When $\omega_{\mathrm{N}}^{\mathrm{tum}} < \omega \leq \omega_{\mathrm{H}}^{\mathrm{tum}}$, the cell becomes hypoxic and pauses its cell cycle until ω increases above this threshold. If $0 \leq \omega \leq \omega_{\mathrm{N}}^{\mathrm{tum}}$, then the tumour cell dies and becomes a necrotic cell. We also account for contact inhibition in our model. If a cell's area falls below a proportion $A_{i\ \mathrm{tum}}^{H}$ of its target area, then its cell cycle pauses until space is available for proliferation. Each tumour cell i has a subcellular variable denoted T_i which tracks its progress through the cell cycle cell as follows:

$$\frac{dT_i}{dt} = \min\left(\mathcal{H}(\omega(\mathbf{x}_i, t) - \omega_{\mathrm{H}}^{\mathrm{tum}}), \mathcal{H}(A_i - A_{i\ \mathrm{tum}}^{H} \pi R_{\mathrm{Cell}}^2)\right). \tag{15}$$

where A_i is the area of the cell calculated as $A_i = \pi r_i^2$ and r_i is the estimated cell radius calculated via the average separation between cells within the interaction radius of i.

Each cell has a target cell cycle duration τ_i, drawn at random from a uniform distribution of $U\left(0.75\tau_{\mathrm{tum}}, 1.25\tau_{\mathrm{tum}}\right)$ with average cell cycle duration τ_{tum}. When $T_i = \tau_i$, cell division occurs and a new cell is placed at a distance half a cell diameter away, in a randomly chosen direction. Both cells are assigned new cell cycle durations. T_i is set to 0 for each cell, and then evolves according to Eq (15). The equilibrium spring length associated with the new cells is set to $s_i = 0.5R_{\mathrm{Cell}}$ to account for their reduced size, and increases linearly over the course of 1 hour until it reaches $s_i = R_{\mathrm{Cell}}$.

Force laws for tumour cells Tumour cell movement is governed by the force balance described in Equation (6). The net force incorporates intercellular interactions as described above, and a chemotactic force due to spatial gradients in EGF. The chemotactic force, denoted $\mathbf{F}_i^{\chi_\epsilon}$, has the form

$$\mathbf{F}_i^{\chi_\epsilon} = \chi_\epsilon^T \frac{\nabla \epsilon}{|\nabla \epsilon|} \tag{16}$$

where the parameter χ_ϵ^T determines tumour cell sensitivity to EGF gradients. The net force used in Equation (6) is therefore

$$\mathbf{F}_i = \mathbf{F}_i^m + \mathbf{F}_i^{\chi_\epsilon}. \tag{17}$$

Stromal cells

Cell cycle and proliferation The same cell cycle model is used for stromal and tumour cells, but it is parameterised differently to account for the increased ability of tumour cells to survive in adverse conditions such as lower oxygen environments or increased mechanical pressure from neighbouring cells. Like tumour cells, stromal cells possess a subcellular age variable T_i which evolves according to the equation

$$\frac{dT_i}{dt} = \min\left(\mathcal{H}(\omega(\mathbf{x}_i, t) - \omega_{\mathrm{H}}^{\mathrm{str}}), \mathcal{H}(A_i - {A_i^H}_{\mathrm{str}} \pi R_{\mathrm{Cell}}^2)\right) \tag{18}$$

where the parameter $\omega_{\mathrm{H}}^{\mathrm{str}}$ determines the oxygen threshold below which stromal cells become hypoxic and the parameter ${A_i^H}_{\mathrm{str}}$defines the proportion of a stromal cell's target area below which the cell cycle stops.

As with tumour cells, we define a second oxygen threshold $\omega_{\mathrm{N}}^{\mathrm{str}}$, below which stromal cells become necrotic. We suppose that $\omega_{\mathrm{H}}^{\mathrm{str}} < \omega_{\mathrm{H}}^{\mathrm{tum}}$ and ${A_i^H}_{\mathrm{str}} < {A_i^H}_{\mathrm{tum}}$ to account for the ability of tumour cells to proliferate in more adverse environments than stromal cells.

Force laws Stromal cells are subject to the same intercellular forces as macrophages and tumour cells, and defined by Equations (7)-(8). The force balance for stromal cells used in Equation (6) is

$$\mathbf{F}_i = \mathbf{F}_i^m. \tag{19}$$

Necrotic cells

When a stromal or tumour cell is marked for cell death, as a result of oxygen starvation or being killed by a macrophage, it irreversibly becomes necrotic. A necrotic cell i occupies space for $\bar{\tau}_i$ hours, where $\bar{\tau}_i$ is drawn from a uniform distribution $U(0.75\,\bar{\tau}, 1.25\,\bar{\tau})$ and $\bar{\tau}$ is the average duration of necrosis. Over this time period, the necrotic cell shrinks in size, by reducing its equilibrium spring constant, s_i, at a constant rate until $s_i = 0$. The cell is then removed from the simulation. While s_i is being reduced, the spring constant μ associated with the cell is also reduced to 0 at a constant rate, to account for weakening of the intercellular forces between degrading necrotic cells and other cells.

Initial and boundary conditions for cells

RDEs describing the diffusible species are solved numerically on a regular triangular mesh with edge length 1 cell diameter. We initialise the simulation by selecting lattice sites to act as point vessels, which do not occupy space or interact directly with cells in our model. All lattice sites more than R_{B} cell diameters from the centre of the domain are possible locations for blood vessels, and N_{B} of these sites are chosen, at random, to be blood vessels. This ensures that the centre of the domain is at least R_{B} cell diameters from the nearest blood vessel and, hence, that there is sufficient space to observe macrophage movement between blood vessels and the tumour, while also ensuring that cells near the domain boundaries are well-oxygenated.

The domain is initialised with stromal cells filling the domain in rows that are 0.75 cell diameters apart, with alternating rows offset by 0.375 cell diameters (i.e., forming a hexagonal lattice). We place four tumour cells in a cluster at the centre of the domain approximately 0.5 cell diameters apart. Stromal and tumour cells are assigned cell cycle durations τ_i from the relevant distributions, and cell cycle progression times T_i selected at random from a uniform distribution $U\left(0, \tau_i\right)$. All cells are constrained to remain within the domain by imposing reflective boundary conditions.

Schematic

The schematic in Fig S0 shows the order in which the above processes occur. After initialisation, on each timestep the concentrations of diffusible species are updated and macrophage extravasation occurs. Individual cell cycles, target spring lengths, phenotype changes, and proliferation are then updated. Finally, cells are moved to their new locations.

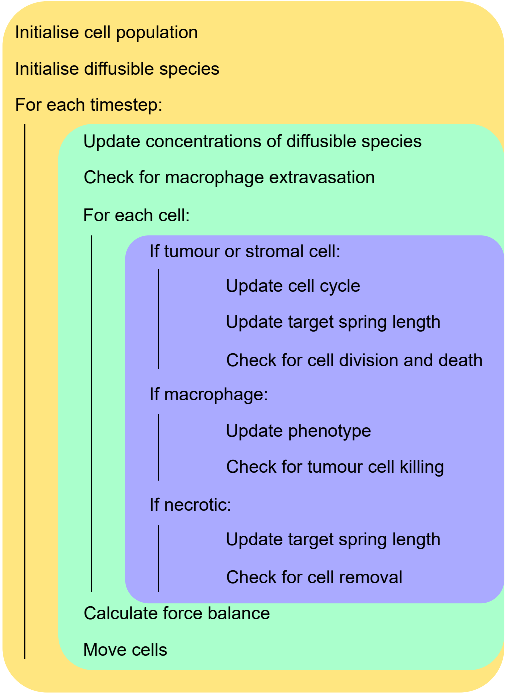

Fig S0. Model overview
Schematic showing an overview of the order in which the model is initialised, updated at each timestep, and updated at a cell level.

Table of ABM parameters

In Table 1 we list the model parameters, their default dimensional and dimensionless values or ranges, and supporting references where these are available. Values of some parameters, indicated with $*$, have been estimated based on model behaviour.

Our model is implemented such that typical scales are given by:

- Length: 1 cell diameter (taken as 20μm) is 1 unit of length.
- Time: 1 hour is 1 unit of time.
- Concentration: the boundary concentration of oxygen is 1.

Table 1. Table of parameters

Parameter	Symbol	Value	Units	Dimensionless value	Reference
Timestep	dt	1/120	hours	1/120	[1]
Damping coefficient	ν	0.4	$N\,s^{-1}m^{-1}$	1	[1, 11, 12]
Blood vessel exclusion radius	R_B	340	μm	17	$*$
Number of blood vessels	N_B	35	-	35	$*$
Radius of interaction	R_{int}	21 - 36	μm	1.5	[12]
Cell radius	R_{Cell}	7 - 12	μm	0.5	[13]
Spring constant	μ	3 - 50	μg Cell diameter^{-1} hours^{-2}	5	[12, 14]
Intercellular adhesion scaling coefficient	α	-	-	5	[1, 12]
Random force coefficient	D	Assumed	Cell diameter^2 hours^{-1}	0.01	[1]
Concentration of oxygen at blood vessels	ω_0	100 - 150	mm Hg	1.0	[15, 16]
Oxygen diffusion coefficient	D_ω	1,750	μm^2 s^{-1}	1.0	[1, 17]
Oxygen consumption rate	κ_ω	20×10^{-18}	mol cell^{-1} s^{-1}	0.03	[1, 17]
Tumour cell hypoxia threshold	ω_H^{tum}	30 - 70	mm Hg	0.01	[1], $*$
Tumour cell necrosis threshold	ω_N^{tum}	10	mm Hg	0.01	[1], $*$
Stromal cell hypoxia threshold	ω_H^{str}	30 - 70	mm Hg	0.1	[1], $*$
Stromal cell necrosis threshold	ω_N^{str}	10	mm Hg	0.01	[1], $*$
CSF-1 diffusion coefficient	D_c	160	μm^2 s^{-1}	1.0	[18], $*$
CSF-1 production rate	κ_c	1.7×10^{-23}	mol m^{-3} s^{-1}	0.25	[18], $*$
CSF-1 decay rate	λ_c	1.9×10^{-4}	s^{-1}	0.02	[18], $*$
TGF-β diffusion coefficient	D_g	21.3	μm^2 s^{-1}	0.1	[19], $*$
TGF-β production rate	κ_g	0.01-0.11	ng (million cells $\times$ day)$^{-1}$	0.1	[19], $*$
TGF-β decay rate	λ_g	0.23-0.34	min^{-1}	0.1	[20], $*$
EGF diffusion coefficient	D_ϵ	160	μm^2 s^{-1}	0.2	[18], $*$
EGF production rate	κ_ϵ	1.7×10^{-23}	mol m^{-3} s^{-1}	0.2	[18], $*$
EGF decay rate	λ_ϵ	1.9×10^{-4}	s^{-1}	0.1	[18], $*$
Concentration of CXCL12 at blood vessels	ξ_0	Assumed	mm Hg	1.0	$*$
CXCL12 diffusion coefficient	D_ξ	150×10^{-6}	μm^2 s^{-1}	1.0	[21], $*$
CXCL12 decay rate	λ_ξ	2×10^{-5}	s^{-1}	0.02	[21], $*$
Chemotaxis sensitivity coefficient (macrophage to CSF-1)	χ_c^m	Assumed	Cell diameter^2 hour^{-1} nM^{-1}	0 - 5	$*$
Chemotaxis sensitivity coefficient (macrophage to CXCL-12)	χ_ξ^m	Assumed	Cell diameter^2 hour^{-1} nM^{-1}	0 - 5	$*$
Chemotaxis sensitivity coefficient (tumour cell to EGF)	χ_ϵ^T	Assumed	Cell diameter^2 hour^{-1} nM^{-1}	0 - 5	$*$
Half-maximal macrophage extravasation CSF-1 concentration	$c_{1/2}$	Assumed	mol Cell diameter^{-3}	0.1 - 0.5	$*$
Maximum probability of macrophage extravasation	$P^\star$	Assumed	-	0.01 - 0.1	$*$
Critical TGF-β threshold	g_{crit}	Assumed	mol Cell diameter^{-3}	0 - 2	$*$
Average duration of tumour cell cycle	τ_{tum}	24	hours	24	[1], $*$
Average duration of stromal cell cycle	τ_{str}	32	hours	32	$*$
Average duration of necrosis	$\bar{\tau}$	48	hours	48	[1], $*$
Proportion of equilibrium area for contact inhibition (tumour cell)	$A_{i\ tum}^H$	Assumed	-	0.6	$*$
Proportion of equilibrium area for contact inhibition (stromal cell)	$A_{i\ str}^H$	Assumed	-	0.75	$*$
Macrophage phenotype increment	Δp	Assumed	hours^{-1}	0.01	$*$
Maximum killing probability per hour	$P_\varphi^\star$	Assumed	-	0.2	$*$
Killing cooldown duration	t_{cool}	Assumed	hours	4	$*$

References

1. Bull JA, Mech F, Quaiser T, Waters SL, Byrne HM. Mathematical modelling reveals cellular dynamics within tumour spheroids. PLOS Computational Biology. 2020;16(8):e1007961. doi:10.1371/journal.pcbi.1007961.

2. Vipond O, Bull JA, Macklin PS, Tillmann U, Pugh CW, Byrne HM, Harrington HA. Multiparameter persistent homology landscapes identify immune cell spatial patterns in tumors. Proceedings of the National Academy of Sciences. 2021;118(41):e2102166118. doi:10.1073/pnas.2102166118.

3. Pitt-Francis J, Pathmanathan P, Bernabeu MO, Bordas R, Cooper J, Fletcher AG, et al. Chaste: A test-driven approach to software development for biological modelling. Computer Physics Communications. 2009;180(12):2452–2471. doi:10.1016/j.cpc.2009.07.019.

4. Mirams GR, Arthurs CJ, Bernabeu MO, Bordas R, Cooper J, Corrias A, et al. Chaste: An Open Source C++ Library for Computational Physiology and Biology. PLoS Computational Biology. 2013;9(3). doi:10.1371/journal.pcbi.1002970.

5. Cooper F, Baker R, Bernabeu M, Bordas R, Bowler L, Bueno-Orovio A, et al. Chaste: Cancer, Heart and Soft Tissue Environment. Journal of Open Source Software. 2020;5(47):1848. doi:10.21105/joss.01848.

6. Arwert EN, Harney AS, Entenberg D, Wang Y, Sahai E, Pollard JW, et al. A Unidirectional Transition from Migratory to Perivascular Macrophage Is Required for Tumor Cell Intravasation. Cell Reports. 2018;23(5):1239–1248. doi:10.1016/j.celrep.2018.04.007.

7. Wyckoff J, Wang W, Lin EY, Wang Y, Pixley F, Stanley ER, et al. A paracrine loop between tumor cells and macrophages is required for tumor cell migration in mammary tumors. Cancer Research. 2004; p. 7022–7029. doi:10.1158/0008-5472.CAN-04-1449.

8. Wyckoff JB, Wang Y, Lin EY, Li JF, Goswami S, Stanley ER, et al. Direct visualization of macrophage-assisted tumor cell intravasation in mammary tumors. Cancer Research. 2007;67(6):2649–2656. doi:10.1158/0008-5472.CAN-06-1823.

9. Drasdo D, Höhme S. A single-cell-based model of tumor growth in vitro: monolayers and spheroids. Physical Biology. 2005;2(3):133–147. doi:10.1088/1478-3975/2/3/001.

10. Meineke FA, Potten CS, Loeffler M. Cell migration and organization in the intestinal crypt using a lattice-free model. Cell Proliferation. 2001;34(4):253–266. doi:10.1046/j.0960-7722.2001.00216.x.

11. Pathmanathan P, Cooper J, Fletcher A, Mirams G, Murray P, Osborne JM, et al. A computational study of discrete mechanical tissue models. Physical biology. 2009;6(3):036001. doi:10.1088/1478-3975/6/3/036001.

12. Osborne JM, Fletcher AG, Pitt-Francis JM, Maini PK, Gavaghan DJ. Comparing individual-based approaches to modelling the self-organization of multicellular tissues. PLoS Computational Biology. 2017;13(2):1–34. doi:10.1371/journal.pcbi.1005387.

13. Laget S, Broncy L, Hormigos K, Dhingra DM, BenMohamed F, Capiod T, et al. Technical insights into highly sensitive isolation and molecular characterization of fixed and live circulating tumor cells for early detection of tumor invasion. vol. 12; 2017.

14. Dunn SJ, Näthke IS, Osborne JM. Computational models reveal a passive mechanism for cell migration in the crypt. PLoS ONE. 2013;8(11):1–18. doi:10.1371/journal.pone.0080516.

15. Mueller-Klieser WF, Sutherland RM. Oxygen tensions in multicell spheroids of two cell lines. British Journal of Cancer. 1982;45(2):256–264. doi:10.1038/bjc.1982.41.

16. Grimes DR, Kelly C, Bloch K, Partridge M. A method for estimating the oxygen consumption rate in multicellular tumour spheroids. Journal of the Royal Society Interface. 2014;11(92). doi:10.1098/rsif.2013.1124.

17. Schaller G, Meyer-Hermann M. Multicellular tumor spheroid in an off-lattice Voronoi-Delaunay cell model. Physical Review E - Statistical, Nonlinear, and Soft Matter Physics. 2005;71(5):1–16. doi:10.1103/PhysRevE.71.051910.

18. Elitas M, Zeinali S. Modeling and Simulation of EGF-CSF-1 pathway to Investigate Glioma - Macrophage Interaction in Brain Tumors. International Journal of Cancer Studies & Research (IJCR). 2016; p. 1–8. doi:10.19070/2167-9118-SI05001

19. Albro MB, Nims RJ, Cigan AD, Yeroushalmi KJ, Alliston T, Hung CT, Ateshian GA. Accumulation of Exogenous Activated TGF-β in the Superficial Zone of Articular Cartilage. Biophysical Journal. 2013;104(8):1794–1804. doi:10.1016/j.bpj.2013.02.052.

20. Wakefield LM, Winokur TS, Hollands RS, Christopherson K, Levinson AD, Sporn MB. Recombinant Latent Transforming Growth Factor β1 Has a Longer Plasma Half-Life in Rats than Active Transforming Growth Factor β1, and a Different Tissue Distribution. The Journal of Clinical Investigation. 1990;86(6):1976–1984. doi:10.1172/JCI114932.

21. Chang SL, Cavnar SP, Luker KE, Takayama S, Luker GD, Linderman JJ. Cell, Isoform, and Environment Factors Shape Gradients and Modulate Chemotaxis. PLoS ONE. 2015;10(4):e0123450. doi:10.1371/journal.pone.0123450.
