# Supplementary material for: Quantification of spatial and phenotypic heterogeneity in an agent-based model of tumour-macrophage interactions
Source: PLoS Comput Biol. 2023 Mar 27;19(3):e1010994. doi: 10.1371/journal.pcbi.1010994 (PMC10079237; doi:10.1371/journal.pcbi.1010994)
Supplement: S2 Appendix — (PDF) [file pcbi.1010994.s002.pdf]

## S2 Appendix: Tumour progression in the presence and absence of macrophages

Fig S2 shows how the presence of macrophages can alter the spatial composition and growth dynamics of a small number of tumour cells initially located at the centre of a two-dimensional square domain that contains stromal cells and a fixed number of static blood vessels. The vessels are randomly distributed in the domain but excluded from a circle of radius $R_\mathrm{B}$ centred on the initial tumour mass. Panels A and C show that, in the absence of macrophages (i.e., setting the maximum probability of extravasation $P^\star = 0$), the tumour increases rapidly in size but remains as a compact mass. At much longer timescales, the tumour evolves to a steady state where the net proliferation rate of oxygen-rich cells on the tumour periphery balances the net death rate of oxygen-starved cells towards the tumour centre.

When macrophage extravasation is active (Fig S2B and Fig S2D), the ABM reproduces the qualitative behaviours outlined in Fig 2 of the main text [1]. At early times ($t \approx 100$), CSF-1 levels are below the threshold for macrophage extravasation and the domain is devoid of macrophages. As the tumour increases in size, more CSF-1 is produced until, eventually, CSF-1 levels at the blood vessels reach the threshold for extravasation of $\mathrm{M}_1$ macrophages ($t \approx 200$). By $t = 300$, some macrophages have infiltrated the tumour mass. Macrophages that have been exposed to sufficient levels of TGF-$\beta$ become $\mathrm{M}_2$ and migrate back towards nearby blood vessels, in response to spatial gradients in CXCL12. The $\mathrm{M}_2$ macrophages also produce EGF which acts as a chemoattractant for the tumour cells. Thus, at $t = 500$ hours, clusters of $\mathrm{M}_2$ macrophages and tumour cells surrounding multiple blood vessels are visible in the domain.

Comparison of Fig S2A and Fig S2B, and Fig S2C and Fig S2D, reveals how the presence of macrophages can transform a tumour from a rapidly growing, compact mass to one that is slower growing and more diffuse. While the summary data presented in Fig S2D provide useful information about the tumour's overall growth dynamics and changing cellular composition, detailed information about its morphology and spatial heterogeneity is lacking. In Fig S3 we present additional statistics generated from the spatial data at $t = 500$. Fig S3B shows the cross-PCF $g_{TB}(r)$ (mean and SD of 10 simulations) for the tumour cells and blood vessels in Fig S3A. There is complete exclusion between blood vessels and tumour cells up to a radius of approximately 5 cell diameters, a lengthscale characterising the minimum distance between the blood vessels and the tumour mass. By comparison, the cross-PCF $g_{TB}(r)$ in Fig S3D quantifies the short-range clustering of tumour cells and blood vessels in Fig S3C. The peak around $r = 1$ indicates close proximity between some blood vessels and tumour cells. The cross-PCFs provide additional information about the strong short-range clustering of macrophages with tumour cells (Fig S3E) and blood vessels (Fig S3F). Macrophages are strongly correlated with tumour cells, particularly at short lengthscales, indicating colocalisation (Fig S3E). There is also strong short-range colocalisation between macrophages and blood vessels (Fig S3F), suggesting the presence of perivascular niches containing macrophages, tumour cells and blood vessels.

The corresponding weighted PCFs, $wPCF(r, P, B)$ and $wPCF(r, P, T)$ (mean of 10 iterations, Fig S3G and Fig S3H) have the signature of an 'Escape' simulation discussed in the main text (Fig 6).

### Steady state dynamics

In this appendix we study the long term behaviour of our ABM in the absence of macrophages. We use the same (default) parameter values used to generate Fig S2, but extend the simulation time to 2000 hours.

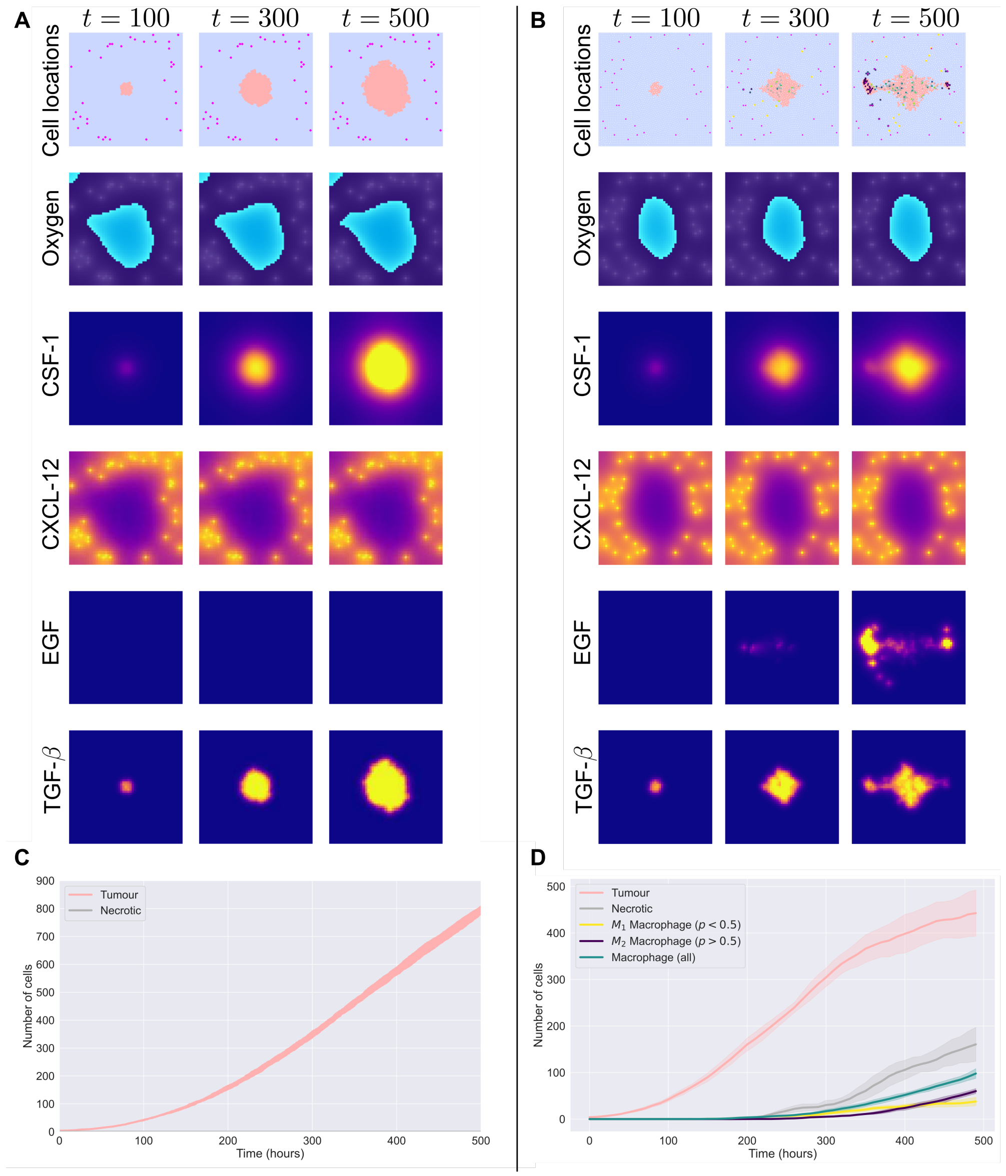


**Fig S2. Representative output from model simulations**
A, B: Spatial distributions of cells, oxygen, CSF-1, CXCL12, EGF and TGF-$\beta$ at times $t = 100, 300, 500$ from simulations which neglect (A, $P^\star = 0$) or include (B, $P^\star = 0.075$) macrophage extravasation. Comparison of these plots shows how the tumour's growth rate and spatial composition can change in the presence of macrophages. Parameter values: $\chi_c^m = 2, \chi_\xi^m = 3$, $\chi_\epsilon^T = 3$, $c_{1/2} = 0.9$, $g_{\text{crit}} = 0.05$; all other parameters fixed at their default values (see S1 Table in S1 Appendix).
C, D: Change in numbers of tumour cells, necrotic cells, $M_1$ and $M_2$ macrophages, and total number of macrophages over time for the simulations presented in (A) and (B) (mean and SD from 10 realisations).

The simulation results presented in Fig S4A show that at long times the tumour evolves to a steady state for which the rate at which oxygen-rich cells on the outer tumour boundary proliferate balances the rate at which necrotic cells in the oxygen-starved core are degraded. We have positioned the blood vessels and fixed the

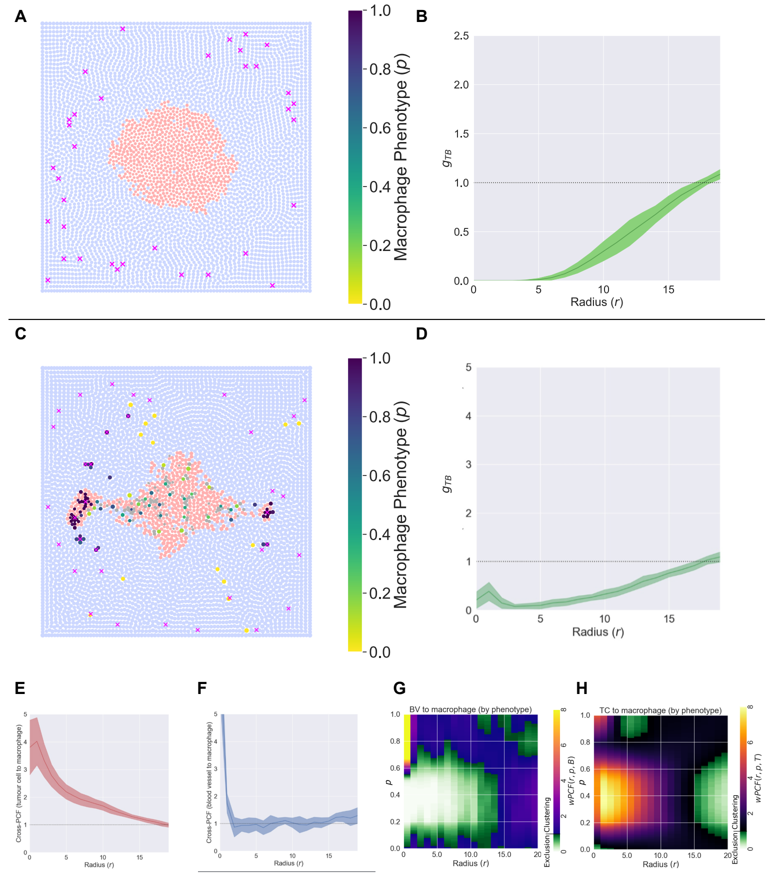


**Fig S3. Statistical analysis of the simulation endpoints in Fig S2**
A: $t = 500$ for tumour growth without macrophage extravasation.
B: The cross-PCF $g_{TB}(r)$ for tumour cells and blood vessels in (A). No tumour cells are observed within a distance of 5 cell diameters from a blood vessel.
C: $t = 500$ for tumour growth with macrophage extravasation.
D: The cross-PCF $g_{TB}(r)$ for the tumour cells and blood vessels in (C). The cross-PCF reveals short range interactions between tumour cells and blood vessels. Comparison with (B) quantifies how the spatial distribution of tumour cells relative to the blood vessels changes in the presence of macrophages.
E: The cross-PCF $g_{TM}(r)$ associated with (C). The cross-PCF reveals strong short range interactions between macrophages and tumour cells.
F: The cross-PCF $g_{BM}(r)$ associated with (C). Short-range correlations between blood vessels and macrophages are very strong and decay rapidly with distance $r$.
G: The weighted PCF $wPCF(r, P, B)$ associated with (C). There is strong, short-range colocalisation of macrophages with $p > 0.6$ and blood vessels, while macrophages with $0.2 \lessapprox p \lessapprox 0.6$ are excluded from regions of radius approximately 10-15 cell diameters surrounding blood vessels.
H: The weighted PCF $wPCF(r, P, T)$ associated with (C). Macrophages with $p > 0.6$ are strongly colocalised with tumour cells at distances $0 \leq r \leq 10$, indicating their presence inside the tumour mass. Short-range colocalisation ($r \approx 3$) is also observed for $M_2$ macrophages with $p \geq 0.9$.

properties of the tumour cells (e.g., the thresholds for hypoxia and necrosis, $\omega_H^{tum}$ and $\omega_N^{tum}$, and the cell cycle duration $\tau_i$) so that a tumour initially located at the centre of

the domain grows as a compact mass. At long times, the total number of tumour cells and necrotic cells remains approximately constant (see Figs S4B and C). Further, the tumour attains its steady state before it can spread to the surrounding blood vessels.

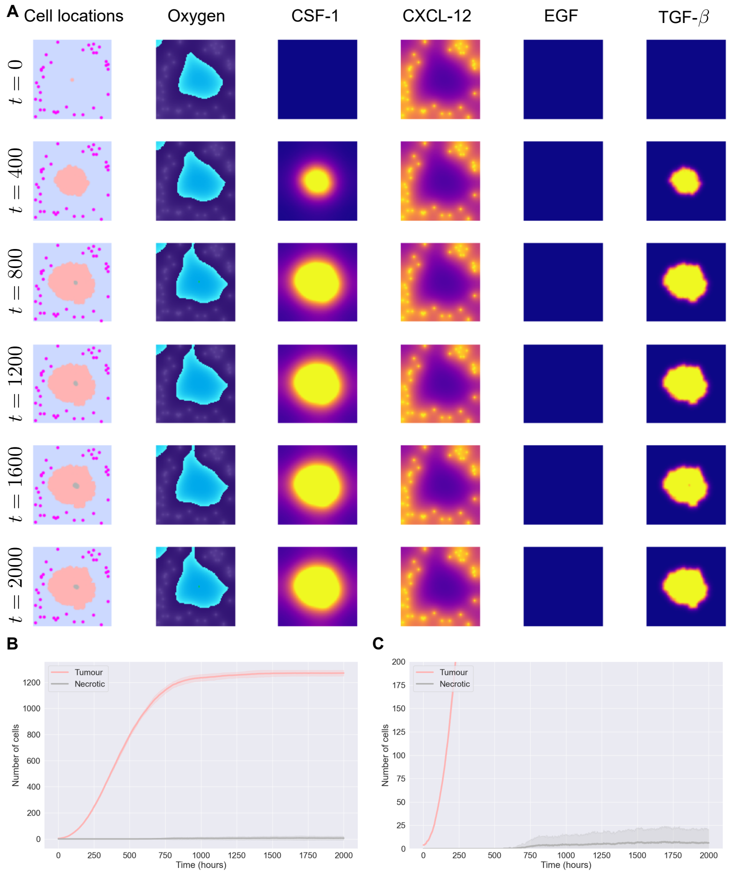


**Fig S4. Tumour growth in the absence of macrophages**
A: When no macrophages enter the simulation, the tumour grows as a compact mass in response to oxygen supplied from blood vessels. At long times, the tumour attains a steady state with a central necrotic core. At steady state, the proliferation rate of cells on the oxygen rich outer tumour boundary balances the death rate of cells in the central, oxygen-starved necrotic core. B/C: Tumour cell counts reach a steady state at approximately $t = 1000$. After approximately $t = 750$ a necrotic core forms due to hypoxia at the tumour centre. (Panel C shows a magnified view of panel B).

## References

1. Arwert EN, Harney AS, Entenberg D, Wang Y, Sahai E, Pollard JW, et al. A Unidirectional Transition from Migratory to Perivascular Macrophage Is Required for Tumor Cell Intravasation. Cell Reports. 2018;23(5):1239–1248. doi:10.1016/j.celrep.2018.04.007.
