# Supplementary material for: Quantification of spatial and phenotypic heterogeneity in an agent-based model of tumour-macrophage interactions
Source: PLoS Comput Biol. 2023 Mar 27;19(3):e1010994. doi: 10.1371/journal.pcbi.1010994 (PMC10079237; doi:10.1371/journal.pcbi.1010994)
Supplement: S3 Appendix — (PDF) [file pcbi.1010994.s003.pdf]

S3 Appendix: Derivation of PCFs and Cross-PCFs from wPCF

In the main text, we use the wPCF to identify correlations between a continuous label (phenotype) and a categorical one (cell type). We now explain how we can extend it to study correlations between two continuous labels. Consider two labels u and v associated with each cell, which may be continuous or discrete. For two target marks U and V, the wPCF in its most general form can be written as:

$$wPCF(r, U, V) = \frac{1}{W_U W_V} \sum_{i=1}^{N} \sum_{j=1}^{N} \frac{A}{A_{r_k}(\mathbf{x}_i)} w_u(U, u_i) w_v(V, v_j) I_{[r_k, r_{k+1})}(|\mathbf{x}_i - \mathbf{x}_j|) \quad (1)$$

where w_u and w_v are weighting functions, and $W_U = \sum_i w_u(U, u_i)$ and $W_V = \sum_i w_v(V, v_i)$ are the total weights associated with each mark. The functional forms of w_u and w_v depend on several factors:

- Are u and v discrete or continuous marks?
- What are the ranges of u and v?
- At what resolution do we wish to identify correlations?

Through appropriate choices of w_U and w_V, Equation (1) reduces to the ordinary PCF, the cross-PCF, or the discrete-continuous form of the wPCF that was introduced in the main text. Table S2 illustrates weighting functions which achieve these different cases.

Table S2. Choices of w which simplify the wPCF. By appropriately choosing w_u and w_v, Equation (1) reduces to the wPCF presented in Equation (11), to the cross-PCF in Equation (9), or to the original definition of the PCF.

Function name	Notation	u: Discrete?	v: Discrete?	w_u	w_v
PCF	$g(r)$	Discrete	Discrete	$w_u(U, u_i) = \Theta(U, u_i)$	$w_v(V, v_i) = \Theta(V, v_i)$ with $u = v$
Cross-PCF	$g_{UV}(r)$	Discrete	Discrete	$w_u(U, u_i) = \Theta(U, u_i)$	$w_v(V, v_i) = \Theta(V, v_i)$
wPCF	$wPCF(r, U, V)$	Continuous	Discrete	$w_u(U, u_i)$	$w_v(V, v_i) = \Theta(V, v_i)$
wPCF	$wPCF(r, U, V)$	Continuous	Continuous	$w_u(U, u_i)$	$w_v(V, v_i)$

We note that there is no requirement that u and v are different marks in the calculation of the wPCF, and that useful information may be gained from calculating, for example, $wPCF(r, U, U)$. This is simply a special case $wPCF(r, U, V)$ in which $u = v$, the the same way as the standard PCF is a special case of the cross-PCF.

As an example, Fig S5 shows the cross-PCFs and wPCFs from Fig 5 in the main text. Through appropriate choice of the weighting function used in the wPCF, the cross-PCFs can be thought of as wPCFs in which the weighting function is piecewise constant as a function of P.

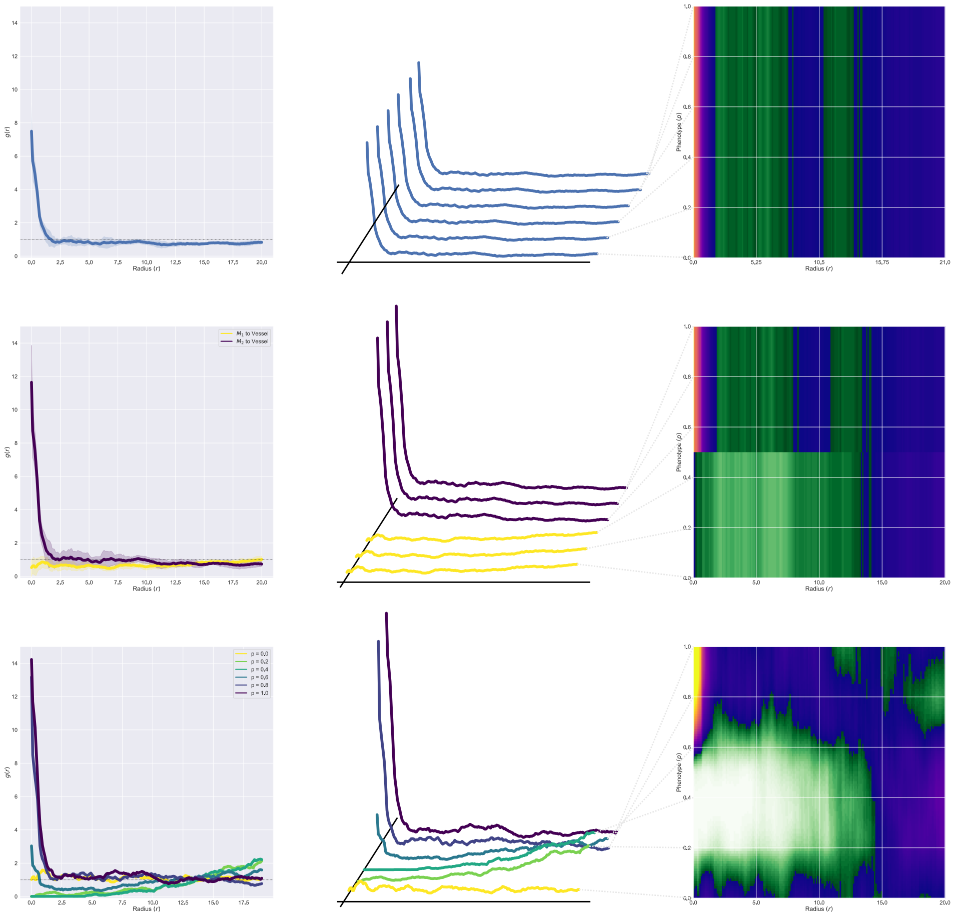

Fig S5. PCFs and Cross-PCFs can be seen as special cases of the wPCF Cross- and wPCFs from Fig 5. By appropriately choosing the weighting function w, the wPCF returns the same results as the cross-PCF.

Top row: $wPCF(r, P, B) = g_{BM}(r)$, with weighting function $w(P, p_i) = 1$ for all target phenotypes P and cell phenotypes p_i.

Middle row: $wPCF(r, P, B) = g_{BM_1}(r)$ for $P \leq 0.5$ and $wPCF(r, P, B) = g_{BM_2}(r)$ for $P > 0.5$, with weighting function $w(P, p_i) = 1$ if P and p_i are both above/below 0.5, and 0 if they are on opposite sides.

Bottom row: standard wPCF as described in Fig 5.
