# Supplementary material for: Quantification of spatial and phenotypic heterogeneity in an agent-based model of tumour-macrophage interactions
Source: PLoS Comput Biol. 2023 Mar 27;19(3):e1010994. doi: 10.1371/journal.pcbi.1010994 (PMC10079237; doi:10.1371/journal.pcbi.1010994)
Supplement: S4 Appendix — (PDF) [file pcbi.1010994.s004.pdf]

S4 Appendix: Comparison of different weighting functions

In this appendix we consider different choices of the weighting, or kernel, function w_p, and apply them to synthetic data. In Fig S6 we place 50 pink crosses uniformly along the line $y = 1$, and 1000 circles according to complete spatial randomness throughout the (2×2) square domain. The circles are labelled with a 'phenotype' p based on their distance from $y = 1$, such that for circle i the label p_i is given by:

$$p_i = \begin{cases} 1 - y_i \text{ if } y_i < 1 \\ 2 - y_i \text{ if } y_i \geq 1 \end{cases} . \tag{1}$$

By construction, this results in two prominent values of p which are correlated with pink crosses at distance r: $p = r$ (below $y = 1$) and $p = 1 - r$ (above $y = 1$).

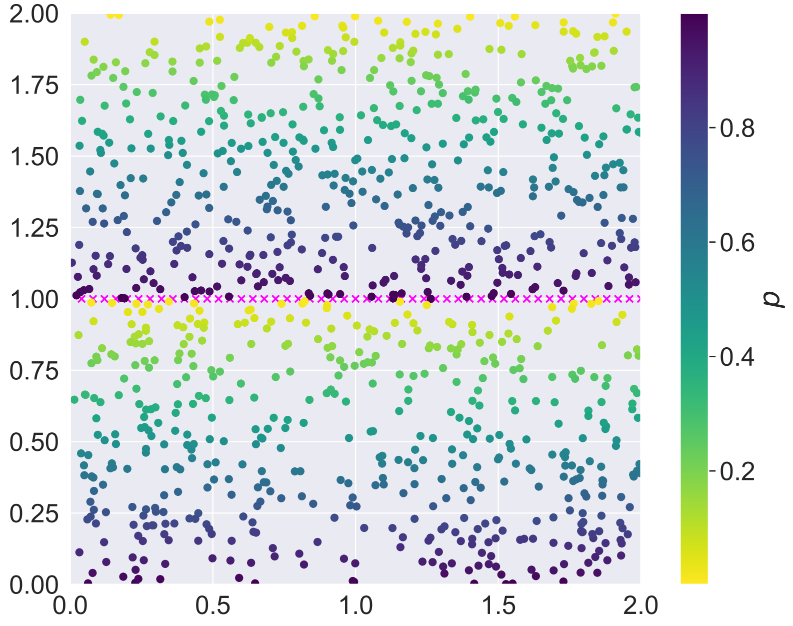

Fig S6. Synthetic data generated to demonstrate different weighting functions
The test data consists of 50 pink crosses ('blood vessels' uniformly positioned along the line $y = 1$) and 1000 circles randomly positioned in the domain ('macrophages' with different phenotypes placed according to complete spatial randomness). Circles are labelled according to their distance from the line $y = 1$.

In Fig S7 we present wPCFs for this point cloud for different choices of the weighting function $w_p(P, p_i)$ in Equation (11). In Fig S7A, we use the kernel from the main text, which has the form $w_p(P, p_i) = \max\left(1 - \frac{|P - p_i|}{\Delta P}, 0\right)$, and consider different values of ΔP. In the main text, we fixed $\Delta P = 0.2$, as it produces a kernel that is sufficiently compact to identify variations in phenotype while being broad enough to reduce noise. In Fig S7B, we fix $w_p(P, p_i) = \max\left(1 - \frac{(P - p_i)^2}{\Delta P}, 0\right)$, which leads to a smoother kernel and, hence, a smoother wPCF. In all cases, we present the wPCF and $w_p(0.5, p_i)$.

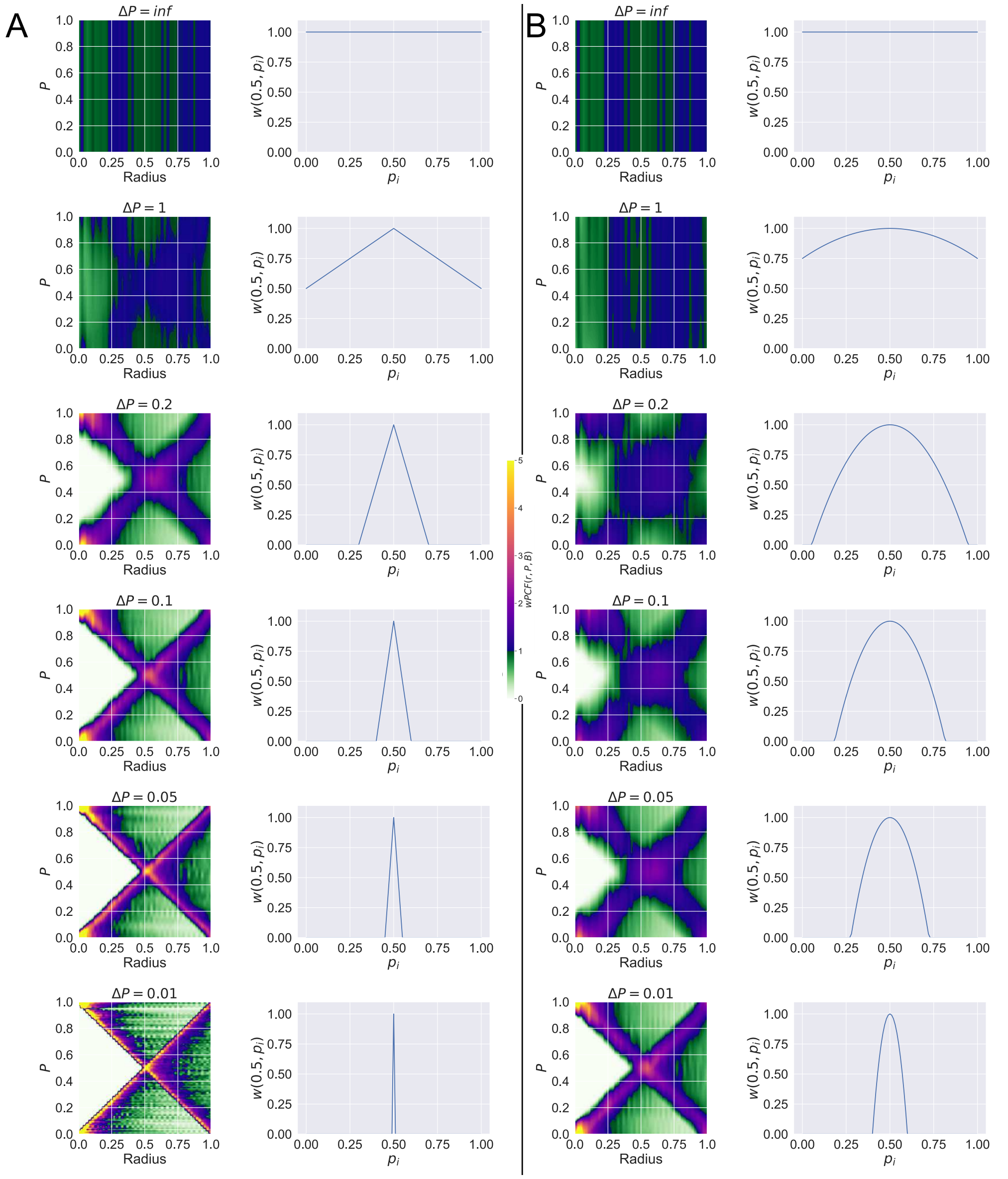

Fig S7. wPCFs generated from different weighting functions $wPCF(r, P, B)$ for the point pattern in Fig S6. Changing the shape of the weighting function adjusts the balance between signal and noise in the wPCF: the narrower the support of w, the more clearly the relationship between label and distance can be discerned. Using a weighting function with extremely narrow support relative to the range of labels results in more noise in the wPCF, most evident in the triangular weighting functions with $\Delta P = 0.05$ and $\Delta P = 0.01$.
A: wPCFs generated using weighting functions of the form $w(P, p) = 1 - m|P - p|$, together with $w(0.5, p)$
B: wPCFs generated using weighting functions of the form $w(P, p) = 1 - m(P - p)^2$, together with $w(0.5, p)$

Fig S7 shows how the shape of the weighting function influences the balance between signal and noise in the wPCF. When the support of the weighting function is broad (e.g., $\Delta P \geq 1$), the wPCF does not identify correlations between r and the target

phenotype P. On the other hand, when the support of the weighting function is narrow (e.g., $\Delta P = 0.01$ for the triangular weighting function), the resulting wPCF identifies correlation well but contains a lot of noise. We conclude that the choice of weighting function can have a strong effect on the resulting wPCF, and should be chosen with care. Selecting a weighting function which is too 'narrow' will result in noisy wPCFs, while one that is too 'broad' will produce wPCFs that are unable to resolve resolution key features. In practice, the appropriate choice is likely to depend on both the distribution of labels in the data and the number of points available, in the same way that the selection of an appropriate annulus radius for the PCF must be tailored to the dataset in question.
