# Supplementary material for: Quantification of spatial and phenotypic heterogeneity in an agent-based model of tumour-macrophage interactions
Source: PLoS Comput Biol. 2023 Mar 27;19(3):e1010994. doi: 10.1371/journal.pcbi.1010994 (PMC10079237; doi:10.1371/journal.pcbi.1010994)
Supplement: S5 Appendix — (PDF) [file pcbi.1010994.s005.pdf]

S5 Appendix: wPCF for comparing two continuous labels

As discussed in S3 Appendix: Derivation of PCFs and Cross-PCFs from wPCF, the wPCF can be generalised to account for relationships between points with two continuous marks (rather than a continuous mark and discrete mark). In its most general form, the PCF is given by

$$wPCF(r, U, V) = \frac{1}{W_U W_V} \sum_{i=1}^{N} \sum_{j=1}^{N} \frac{A}{A_{r_k}(\mathbf{x}_i)} w_u(U, u_i) w_v(V, v_j) I_{[r_k, r_{k+1})}(|\mathbf{x}_i - \mathbf{x}_j|) \quad (1)$$

where w_u and w_v are appropriately chosen weighting functions, and $W_U = \sum_i w_u(U, u_i)$ and $W_V = \sum_i w_v(V, v_i)$ are the total weights associated with each mark.

Demonstration on CSR with correlated labels

To demonstrate this, we consider the synthetic point pattern in Fig S8A. There are two different types of points: 200 circles with a continuous label $p \in [0, 1]$, and 200 triangles with a continuous label $\psi \in [0, 10]$, with locations distributed according to complete spatial randomness. The scales of the labels are chosen to demonstrate the ability of the wPCF to analyse marks which vary over different ranges. The location (x_i, y_i) of each point is chosen randomly, but their labels are defined as follows: $p_i = y_i$ and $\psi_i = 10(1 - y_i)$.

We expect to see colocalisation of triangles with label ψ and circles with label $p = 1 - 0.1\psi$. More generally, we expect to observe strong correlation at distance r between triangles with a target label Ψ and circles with a target label $P = 1 - 0.1\Psi \pm r$.

Since we must now specify two target labels, $p = P$ and $\psi = \Psi$, and a radius, r, $wPCF(r, P, \Psi)$ is a three dimensional statistic. This can be most easily visualised by considering fixed values of r, and observing which values of P and Ψ lead to higher or lower values of the wPCF. Figs S8B-D show $wPCF(r, P, \Psi)$ for $r = 0, 0.25, 0.5$. The dotted black lines in each panel represent the lines $P = 1 - 0.1\Psi \pm r$, and show that the wPCF successfully describes the relationships between points with label P and Ψ separated by distance r.

In this example, we take $w_p(P, p_i) = \max\left(1 - \frac{|P - p_i|}{\Delta P}, 0\right)$ with $\Delta P = 0.1$, and $w_\psi(\Psi, \psi_i) = \max\left(1 - \frac{|\Psi - \psi_i|}{\Delta \Psi}, 0\right)$ with $\Delta\Psi = 1$, to reflect the fact that the range of ψ is 10 times larger than that of p.

Demonstration on data from Fig 6

Figs S9 and S10 show correlations between macrophages with phenotype p_A and those with phenotype p_B in the simulation endpoints shown in Fig 6 (averaged over 10 repetitions).

Fig S9 shows $wPCF(r = 0, p_A, p_B)$. In each case, macrophages with similar phenotypes are observed within distance $r \in [0, 1]$ much more often than spatial randomness would suggest, indicating that similar macrophages are frequently found together. We highlight two features of the simulations highlighted by $wPCF(r = 0, p_A, p_B)$. Firstly, in panel B we see a peak at $p_A = 0.8, p_B = 0.4$ (and vice versa). This shows colocation between macrophages which are at the M_2 end of the phenotype spectrum and macrophages which have begun to transition. Secondly, in panel C we see strong anti-correlation between M_1 and M_2 macrophages, emphasising that they preferentially localise in different areas of the tissue.

Fig S10 shows an alternative way of visualising $wPCF(r, p_A, p_B)$, in which we fix specific values of p_A and p_B in order to visualise correlations at different lengthscales.

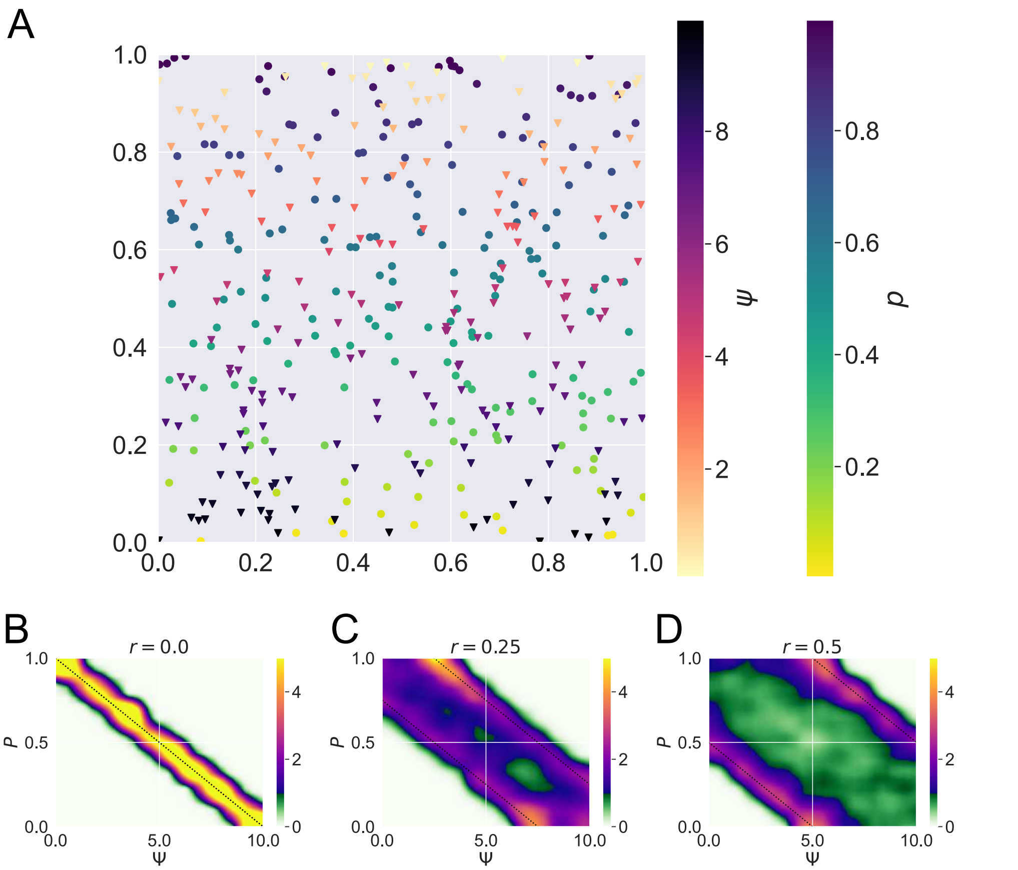

Fig S8. wPCF comparing two continuous labels
A: Two types of points (circles with label p and triangles with label ψ) distributed at random. Points are labelled according to their y coordinate, with point i having $p_i = y_i$ or $\psi_i = 10(1 - y_i)$.
B-D: $wPCF(r, P, \Psi)$ for B) $r = 0$, C) $r = 0.25$, D) $r = 0.5$. Dotted black lines show the expected maximal values of the wPCF according to the construction of the point pattern.

We note that the scale of the wPCF here (in some places $wPCF > 50$) suggests that these effects are likely to be strongly influenced by individual cells, since there are only small numbers of macrophages present with some combinations of p_A and p_B.

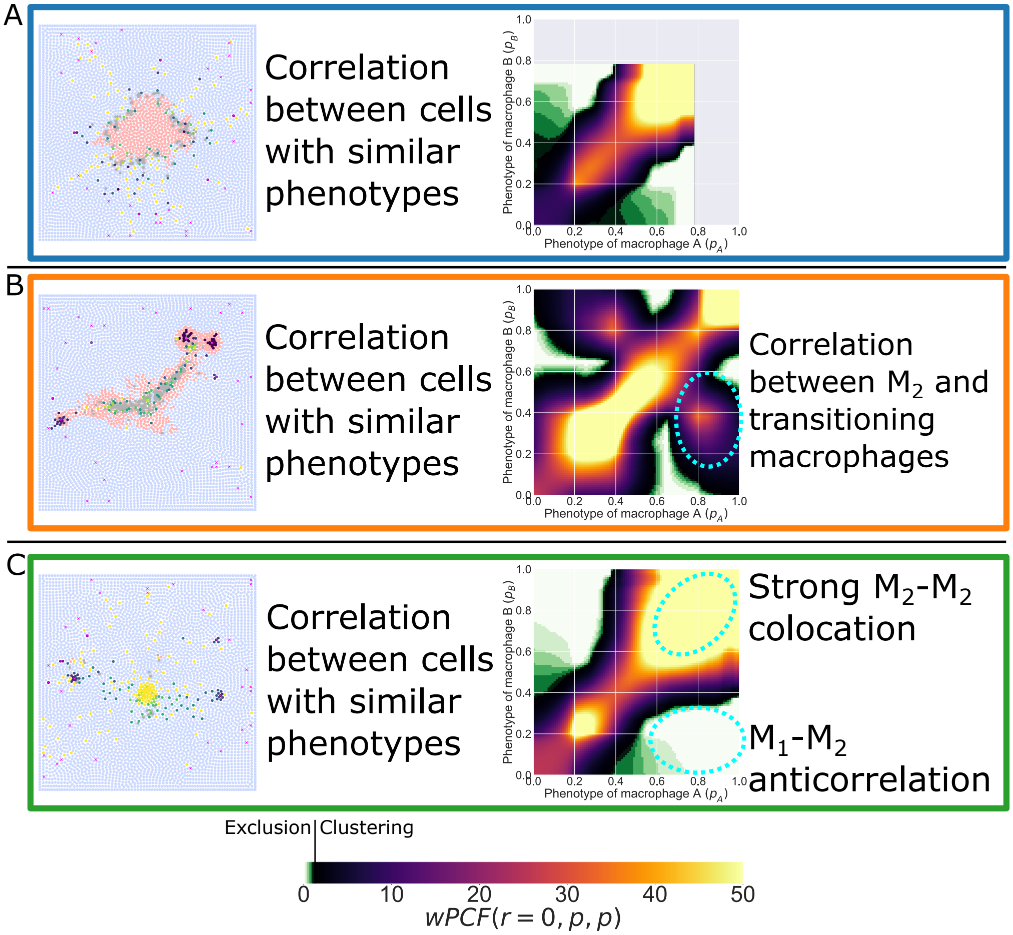

Fig S9. wPCF comparing two continuous labels
$wPCF(r = 0, p_A, p_B)$ for simulations in Fig 6. These plots show clustering between macrophages with phenotype p_A and p_B at short distances ($r \in [0, 1]$).

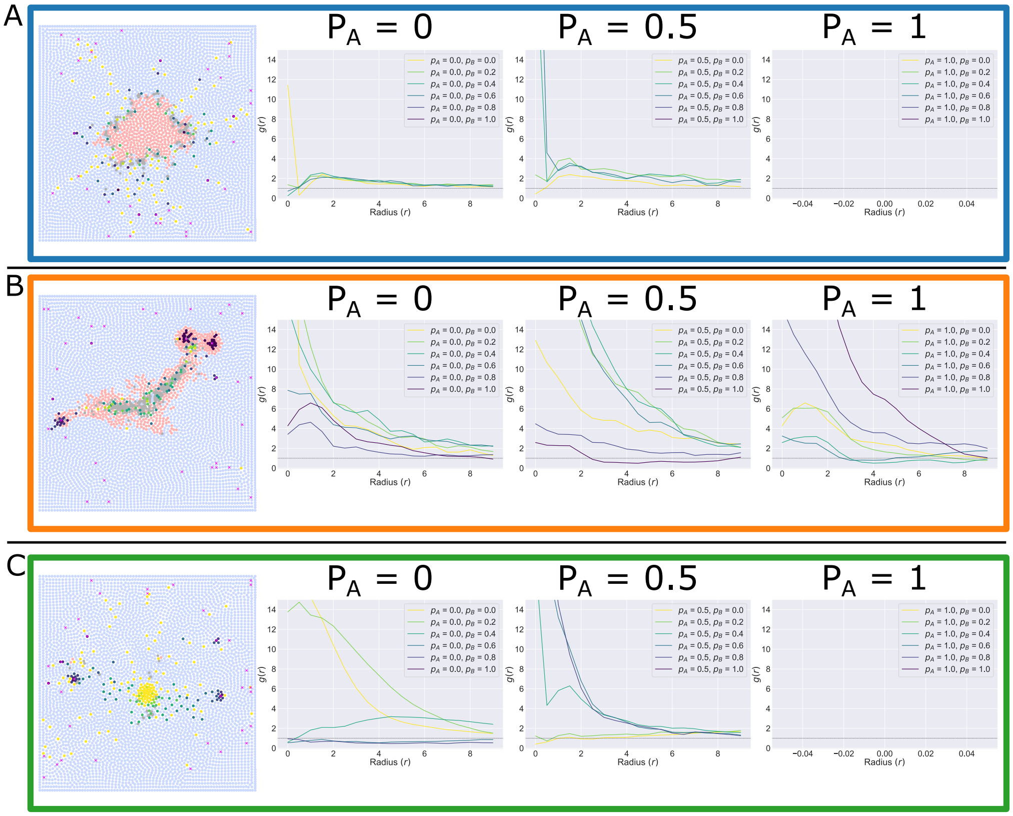

Fig S10. wPCF comparing two continuous labels
$wPCF(r, p_A, p_B)$ for fixed values $p_A \in [0, 0.5, 1]$ and $p_B \in [0, 0.2, 0.4, 0.6, 0.8, 1]$. As in Fig 3, visualising the wPCF for fixed values of p_A and p_B leads to lines with the familiar interpretation of a cross-PCF.
