# Supplementary material for: Quantification of spatial and phenotypic heterogeneity in an agent-based model of tumour-macrophage interactions
Source: PLoS Comput Biol. 2023 Mar 27;19(3):e1010994. doi: 10.1371/journal.pcbi.1010994 (PMC10079237; doi:10.1371/journal.pcbi.1010994)
Supplement: S6 Appendix — (PDF) [file pcbi.1010994.s006.pdf]

S6 Appendix: Vectorisation and Support Vector Machine classification

In this Section we explain how we vectorise $g_{BM}(r)$, $wPCF(r,p,B)$ and $wPCF(r,p,T)$ to define the PCF signature used in this paper. We also consider how the details of the vectorisation (in particular, its granularity) influence the predictions of SVM classification.

Discretisation process

In order to represent the PCF signature as a single vector, we first discretise $g_{BM}(r)$, $wPCF(r,p,B)$ and $wPCF(r,p,T)$. Since we are not aiming to create a classifier with application beyond the proof-of-concept described in this paper, we have not optimised the SVM classification; we have, instead, chosen simple discretisations of the cross-PCF and wPCF based on their numerical calculations. We discretised r by choosing values r_i where $r_i = i\Delta r$ for $i \in [0, ..., 190]$ and $\Delta r = 0.1$ (giving a high-resolution cross-PCF calculated for annuli with inner radius up to 19 cell diameters). Similarly, p is discretised by choosing values $p_i = i\Delta p$ for $i \in [0, ..., 100]$ and $\Delta p = 0.01$, giving a high resolution of p for phenotypes in the range from 0 to 1. This gives 191 values for $g_{BM}(r_i)$, and a further $191 \times 101 = 19291$ values for each of $wPCF(r_i, p_i, B)$ and $wPCF(r_i, p_i, T)$. A single 38,773 dimensional vector $\mathbf{z}$ is then formed by concatenating the discretised and vectorised cross-PCF and wPCFs:

$$\mathbf{z} = \Bigg(g_{BM}(\mathbf{r}), wPCF(\mathbf{r}, p_0, B), ..., wPCF(\mathbf{r}, p_{100}, B), wPCF(\mathbf{r}, p_0, T), ..., wPCF(\mathbf{r}, p_{100}, T) \Bigg).$$

where $(f(\mathbf{r})) = (f(r_0), ..., f(r_{190}))$ and f represents either the cross-PCF or wPCF.

The results presented in Fig S11 show how the classification accuracy of the SVM changes as we vary Δr and Δp and, hence, the resolution of the vectorised PCF signature. We observe that changing the values of Δr and Δp does not substantially alter the accuracy of the SVM described in the main text (where $\Delta r = 0.1$ and $\Delta p = 0.01$). Here, we subsampled the high resolution PCF signatures used in the main text to generate coarser grained PCF signatures with $\Delta r \in [0.1, ..., 1]$ and $\Delta p \in [0.01, 0.05, 0.1, ..., 0.5]$. The resulting vectorised PCF signatures have lower dimensionality than the one described in the main text (38,773), with the most coarsely grained signature having dimensionality of 140. Training an SVM for these lower-dimensional signatures (following the method described in the main text) yields similar overall classification accuracies as the highest-resolution signature. We indicate the classifiers with the best (solid black border) and worst (dashed black border) accuracy, as well as the classifier used in the main text (black circle).

Varying the number of training images

We test the effect of the number of training images on the accuracy of the SVM classifier by using subsamples of the 371 images from the training dataset to generate new SVM classifiers. We vary the number of training images sampled from 100 to 370 in steps of 5, and measure the overall classifier accuracy. For each number of training images, we repeat the process for 25 subsamples. The classifier accuracy increases with larger sample sizes, but remains above 70% for all samples. Fig S12 shows the mean and standard deviation of the overall classifier accuracy as the number of images used for training increases.

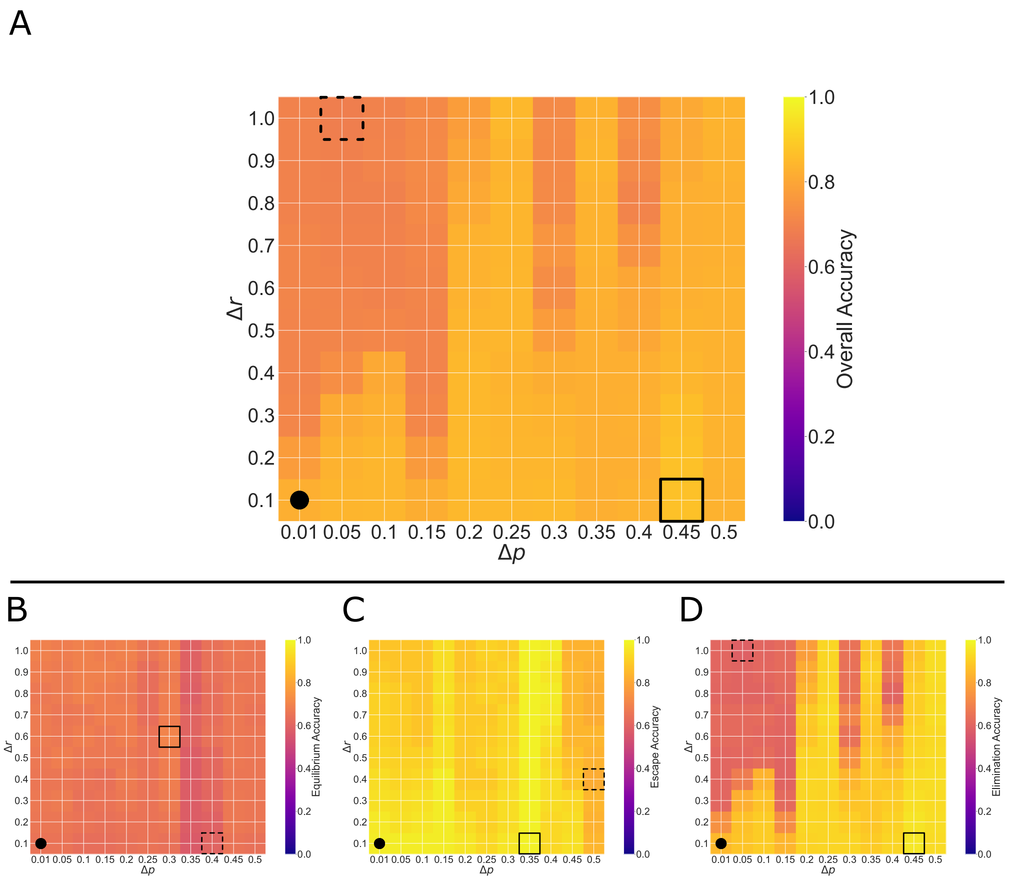

Fig S11. Impact of PCF signature resolution on classification accuracy of the SVM.

The classification accuracy does not change significantly as the spatial and phenotypic resolution (i.e., Δr and Δp) and, hence, the dimensionality of the PCF signature vary. Panel A shows the overall accuracy of the classifier, while Panels B-D show the classification accuracy for Equilibrium, Escape and Elimination simulations respectively. In each panel, we highlight the default values used in the main text (black circle), the maximum accuracy (solid black outline) and the minimum accuracy (dashed black outline).

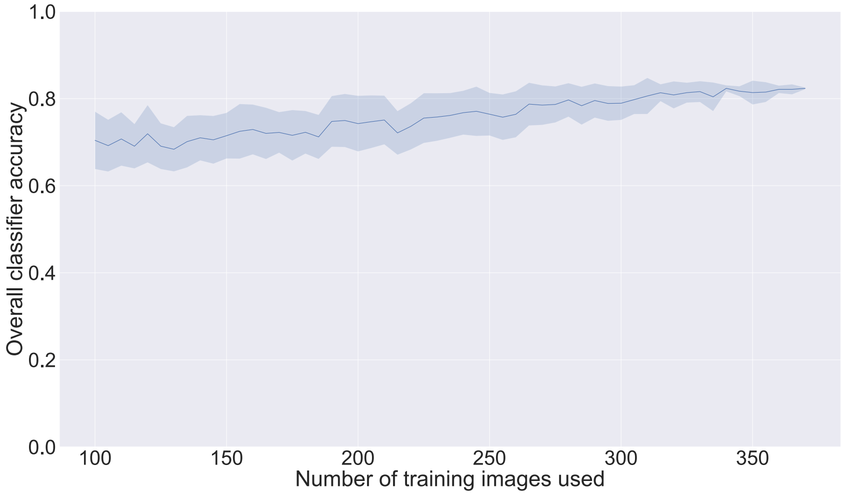

Fig S12. Classification accuracy as a function of the number of training images used.
Increasing the number of training images used in a classifier results in higher classification accuracy. Shaded area shows the standard deviation of 25 subsamples from the original 371 training images.
