# Supplementary material for: Quantification of spatial and phenotypic heterogeneity in an agent-based model of tumour-macrophage interactions
Source: PLoS Comput Biol. 2023 Mar 27;19(3):e1010994. doi: 10.1371/journal.pcbi.1010994 (PMC10079237; doi:10.1371/journal.pcbi.1010994)
Supplement: S7 Appendix — (PDF) [file pcbi.1010994.s007.pdf]

S7 Appendix: Variation in simulation behaviour for a wider range of parameters

In this appendix we present examples of behaviours generated by the ABM when a wider range of parameters are varied. These represent simulations from the second parameter sweep described in the main text, in which 6 parameter values (χ_c^m, χ_ξ^m, χ_ϵ^T, $c_{1/2}$, $P^\star$, and g_{crit}) were chosen uniformly at random. Fig S13 shows some examples of simulations in which macrophage localisation is more diverse than in the examples seen in the 2-parameter sweep.

Panel	χ_c^m	χ_ξ^m	χ_ϵ^T	$c_{1/2}$	$P^\star$	g_{crit}
A	3.494	0.120	2.421	0.409	0.088	0.032
B	2.731	0.190	4.784	0.458	0.011	0.919
C	2.613	0.328	3.442	0.470	0.056	0.955
D	3.448	0.871	3.672	0.497	0.044	0.737
E	2.942	4.074	3.404	0.356	0.011	0.517
F	1.750	3.455	1.892	0.324	0.091	0.032

Table S3. Parameters used for simulations in Fig S13 Values of the six parameters randomly selected for each simulation are shown here; other parameters are fixed at the default values given in S1 Appendix

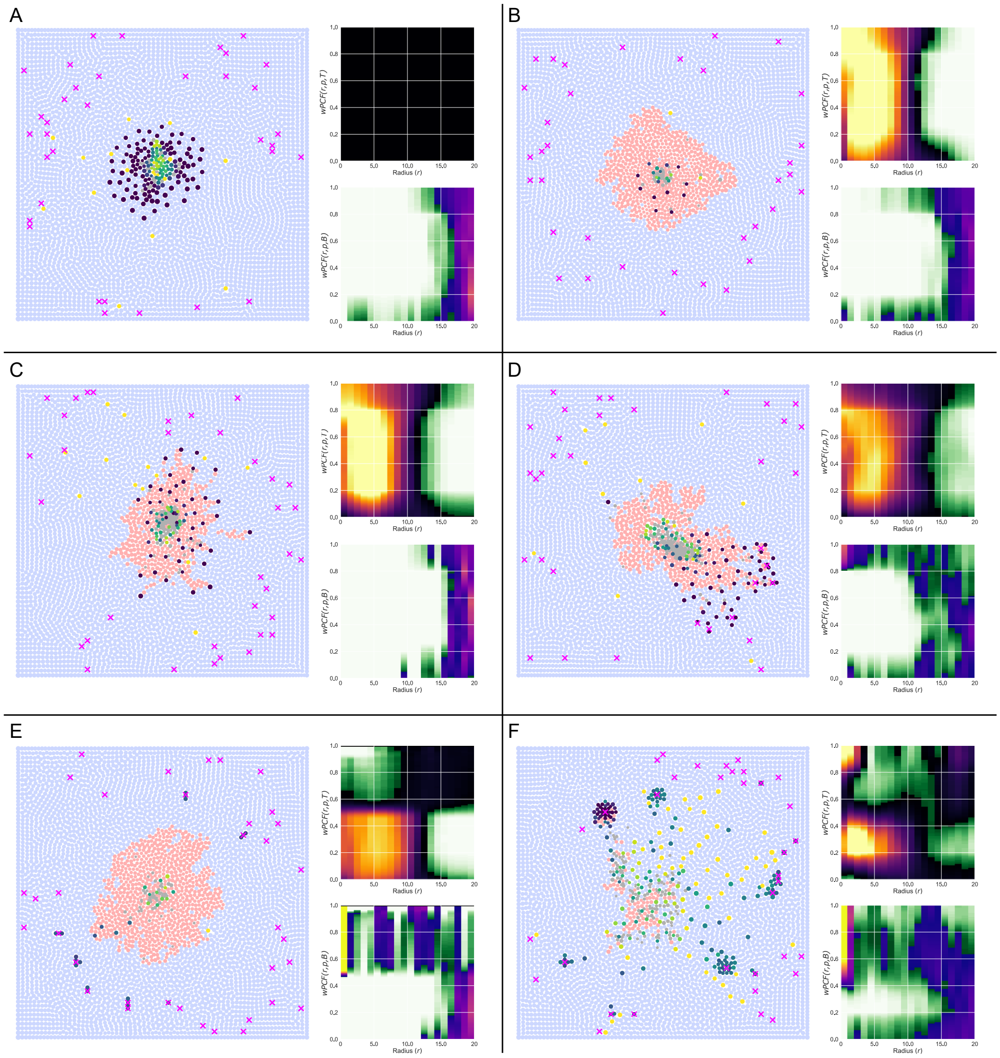

Fig S13. wPCFs distinguish diverse model behaviours

The parameter values used to generate the simulations shown in panels A-F are shown in Table S3. The model generates a wide range of simulation behaviours. Panels show cell locations at $t = 500$, with insets of $wPCF(r, P, T)$ (top) and $wPCF(r, P, B)$ (bottom).

A: Tumour elimination in which M_2-like macrophages do not localise around blood vessels.

B: Compact tumour growth in which M_2-like macrophages remain localised within the tumour mass.

C: Diffuse tumour growth in which M_2-like macrophages direct the migration of streams of tumour cells away from the tumour mass.

D: Diffuse tumour growth in which a large cluster of M_2-like macrophages directs tumour cells to localise around blood vessels.

E: M_2-like macrophages migrate towards blood vessels with a low phenotype ($p \approx 0.5$) and hence do not recruit tumour cells.

F: M_2-like macrophages with phenotypes in the range $0.5 < p \leq 1$ localise around blood vessels, and are accompanied by tumour cells only if the perivascular macrophages have $p \approx 1$.
